# Neto-Mediated Intracellular Interactions Shape Postsynaptic Composition at the *Drosophila* Neuromuscular Junction

**DOI:** 10.1371/journal.pgen.1005191

**Published:** 2015-04-23

**Authors:** Cathy I. Ramos, Oghomwen Igiesuorobo, Qi Wang, Mihaela Serpe

**Affiliations:** Program in Cellular Regulation and Metabolism, NICHD, NIH, Bethesda, Maryland, United States of America; University of Southern California, UNITED STATES

## Abstract

The molecular mechanisms controlling the subunit composition of glutamate receptors are crucial for the formation of neural circuits and for the long-term plasticity underlying learning and memory. Here we use the *Drosophila* neuromuscular junction (NMJ) to examine how specific receptor subtypes are recruited and stabilized at synaptic locations. In flies, clustering of ionotropic glutamate receptors (iGluRs) requires Neto (Neuropillin and Tolloid-like), a highly conserved auxiliary subunit that is essential for NMJ assembly and development. *Drosophila neto* encodes two isoforms, Neto-α and Neto-β, with common extracellular parts and distinct cytoplasmic domains. Mutations that specifically eliminate Neto-β or its intracellular domain were generated. When Neto-β is missing or is truncated, the larval NMJs show profound changes in the subtype composition of iGluRs due to reduced synaptic accumulation of the GluRIIA subunit. Furthermore, *neto-β* mutant NMJs fail to accumulate p21-activated kinase (PAK), a critical postsynaptic component implicated in the synaptic stabilization of GluRIIA. Muscle expression of either Neto-α or Neto-β rescued the synaptic transmission at *neto* null NMJs, indicating that Neto conserved domains mediate iGluRs clustering. However, only Neto-β restored PAK synaptic accumulation at *neto* null NMJs. Thus, Neto engages in intracellular interactions that regulate the iGluR subtype composition by preferentially recruiting and/or stabilizing selective receptor subtypes.

## Author Summary

Ionotropic receptors assembled from different subunits have strikingly different properties and uses. In mammalian brain, the molecular mechanisms controlling the subunit composition of glutamate receptors are critical for the formation of neural circuits and for the long-term plasticity underlying learning and memory. Here we investigate how subunit composition is regulated at the *Drosophila* neuromuscular junction (NMJ), a synapse similar in composition and physiology to mammalian AMPA/Kainate synapses. We find that an auxiliary protein, Neto, which is essential for functional receptors, has a key role in controlling which flavor of glutamate receptors will be at the synapses. In flies, synapse strength and plasticity is modulated by the interplay between two receptor subtypes, A and B. Mutations that eliminate or truncate the Neto-β isoform fail to accumulate the type-A receptors, as well as other postsynaptic proteins important for the synaptic stabilization of type-A receptors. This result indicates that Neto may use its cytoplasmic domains as signaling hubs and organizing platforms to sculpt postsynaptic composition. Neto proteins modulate the formation and function of glutamatergic synapses from worms to humans. Our findings expand the repertoire of Neto proteins and illustrate the richness in synapse modulation brought about by the growing family of auxiliary proteins.

## Introduction

Ionotropic glutamate receptors (iGluRs) play major roles in excitatory transmission in the vertebrate brain and at the insect neuromuscular junction (NMJ). The synapse properties are primarily shaped by the subunit composition of the receptors, which could be further modified by RNA editing and alternative splicing [1]. Changes in the subunit composition of postsynaptic iGluRs, in particular the AMPA-type receptors, have a tremendous impact on the development and plasticity of glutamatergic synapses [2,3]. Mechanisms controlling the recruitment of selective receptor subtypes exist and they integrate signals from multiple signaling pathways and regulate synaptic trafficking via posttranslational modifications within the iGluRs intracellular domains [4,5]. In addition, several auxiliary subunits, which primarily modulate the channel properties, have been implicated in the subcellular distribution of receptors [6]. Whether and how the auxiliary subunits modulate the subunit composition of iGluRs remains unclear.

The *Drosophila* NMJ is a glutamatergic synapse similar to mammalian central synapses. In flies, the iGluRs are heterotetrameric complexes composed of three shared subunits, GluRIIC, GluRIID and GluRIIE, and either GluRIIA (type-A receptors) or GluRIIB (type-B) [7–11]. The function of the fly NMJ also requires Neto (Neuropillin and Tolloid-like), an obligatory auxiliary subunit of the iGluR complexes [12]. In the absence of Neto, or any of the shared iGluR subunits (or GluRIIA and GluRIIB together) the receptors fail to cluster at synaptic locations and the animals die as paralyzed embryos unable to develop into larval stages [12,13]. Genetic manipulation of Neto and iGluR levels indicated that Neto and the shared iGluR subunits are limiting for the synaptic localization of receptors and that GluRIIA and GluRIIB compete with each other for synaptic localization [9,14].

The type-A and type-B receptors differ in their single-channel properties, synaptic currents, regulation by second messenger, and sub-synaptic distribution [13]. The type-B channel desensitizes ten times faster than the type-A [8]. Also, the postsynaptic response to the fusion of single synaptic vesicles (the quantal size) is much reduced when only the type-B receptors are present; in fact, the dose of synaptic GluRIIA versus GluRIIB is a key determinant of quantal size. The interplay between the type-A and type-B synaptic receptors modulates synapse strength and plasticity.

The synaptic accumulation of type-A and type-B receptors at the fly NMJ is differentially regulated. Two hybrid and genetics screens identified Coracle, the *Drosophila* homologue of the mammalian cytoskeletal protein 4.1, as a direct binding partner for the GluRIIA cytoplasmic domain [15]. Disruptions of the actin cytoskeleton or mutations in *coracle* caused selective reduction in GluRIIA synaptic accumulation, suggesting that Coracle anchors the GluRIIA to the actin cytoskeleton. Mutations in the p21-activated kinase (PAK) also disrupt the localization and function of the GluRIIA. PAK co-localizes with the iGluR complexes at postsynaptic densities (PSDs) and, in conjunction with the guanine nucleotide exchange factor Pix and the adaptor protein Dreadlocks, promotes the synaptic accumulation of GluRIIA [16,17]. The synaptic accumulation of GluRIIB appears reduced in mutants of the PDZ (PSD-95/Dlg/Zona occludens-1) domain-containing scaffolding protein Discs large (Dlg) [18]. However, there is no evidence for a direct interaction between GluRIIB and Dlg, and except for GluRIIC [9], the iGluR subunits of the *Drosophila* NMJ lack a PDZ binding domain, suggesting that Dlg influences the synaptic abundance of GluRIIB indirectly. Also, Dlg localizes perisynaptically, at subsynaptic reticulum (SSR) [19]. In contrast, mammalian PSD-95 is a major component of the PSD, and it directly binds and modulates the synaptic targeting of iGluRs [20] and some of their interacting partners, such as the 4.1N protein [21]. The PSD-95-mediated interactions can be further regulated by post-translational modifications such as palmitoylation and phosphorylation [5].

Here we show that Neto is key in regulating the subtype composition of iGluRs at the *Drosophila* NMJ. We characterized a novel Neto isoform, Neto-β, which appears to be the predominant isoform at the fly NMJ. The two *Drosophila* isoforms, Neto-α and Neto-β, share the highly conserved extracellular and transmembrane domains, the hallmark of the Neto family of proteins [6], but have distinct cytoplasmic domains, rich in protein interaction motifs. We generated *neto-β* isoform specific mutants and found that the synaptic accumulation of type-A receptors is selectively impaired at *neto-β* mutant NMJs. Furthermore, Neto-β-deficient synapses fail to recruit PAK, a critical PSD signaling molecule required for the synaptic stabilization of type-A receptors and for the recruitment of other postsynaptic components. Muscle overexpression of either Neto-α or Neto-β isoforms could rescue the lethality and NMJ function of *neto* null mutants, indicating that the shared domains of Neto function to mediate iGluRs clustering. However, only Neto-β could restore the recruitment of PAK at *neto* null synapses. Our data demonstrate that Neto-mediated cytoplasmic interactions control the subtype composition of iGluRs and shape postsynaptic composition.

## Results

### Neto-β, a novel Neto isoform at the *Drosophila* NMJ

**Fig 1:**
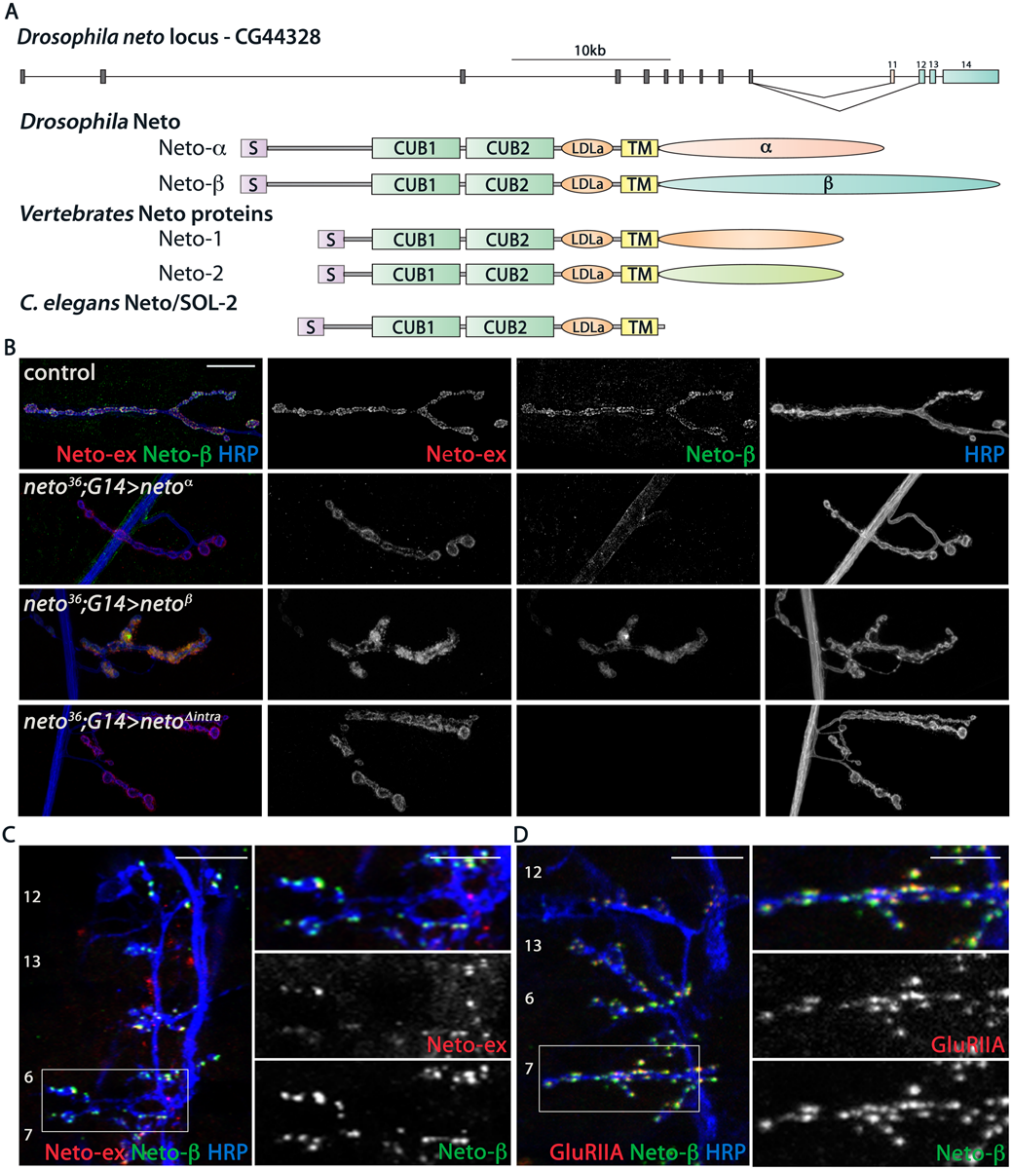
Neto-β is a novel Neto isoform at the *Drosophila* NMJ. (A) Diagram of the *Drosophila neto* locus. Two isoforms are generated by alternative splicing, Neto-α and Neto-β. They have different cytoplasmic domains, but share highly conserved domains, CUB (complement C1r/C1s, Uegf, BMP1), LDLa (LDL receptor class a) and transmembrane (TM), with Neto proteins from vertebrates and *C*. *elegans*. (B) Confocal images of third instar larvae NMJ4 from *neto* ^*null*^ animals rescued with *neto*-*α* (*neto* ^*36*^;*G14>neto* ^*α*^), *neto*-*β* (*neto* ^*36*^;*G14>neto* ^β^), and a *neto* transgene lacking any intracellular part, *neto*Δ^intra^ *(neto* ^*36*^;*G14>neto*Δ^intra^). These Neto variants can rescue the viability and NMJ development in *neto* ^*null*^ animals. The Neto-ex signals mark all NMJs, but the Neto-β antibodies specifically label control and *neto-β* rescued NMJs. (C-D) Confocal images of late embryos ventral muscle fields (indicated in white) labeled for (C) Neto-ex (red), Neto-β (green), HRP (blue) and (D) GluRIIA (red), Neto-β (green), HRP (blue) indicating the presence of Neto-β in the early stages of the larvae development. Genotypes: control (*w* ^*1118*^); *neto* ^*36*^;*G14>neto* ^*α*^ (*neto* ^*36*^ */Y; G14-Gal4/UAS-neto-A9*); *neto* ^*36*^;*G14>neto* ^β^ (*neto* ^*36*^, *UAS-neto-B6/Y; G14-Gal4/+*); *neto* ^*36*^;*G14>neto*Δ^intra^(*neto* ^*36*^ */Y; G14-Gal4/ UAS-neto*Δ^intra^ *-H4*). Scale bars: (B) 20 μm (C-D) 10 μm; 5 μm in details.

*Drosophila neto* codes for two transcripts, cDNA references GH11189 and RE42119. They share the first 10 exons, encoding the extracellular and transmembrane part of Neto, but have alternative exons that generate different intracellular domains (Fig 1A). Exon 11 encodes the cytoplasmic domain of Neto-α, a 206-residue acidic domain (pI 3.88). Exons 12–14 encode the 351-residue Neto-β intracellular domain (pI 8.90). The two domains are predicted to contain multiple phosphorylation and protein interaction motifs (Neto-β shown in S1 Fig), but they show no homology with other Neto proteins. In fact, unlike the highly conserved extracellular and transmembrane domains of Neto proteins, the intracellular parts are highly divergent or even missing, such as in the *C*. *elegans* Neto (Fig 1A) [22–24].

Previous RNA-Seq analyses indicated that *neto-α* and *neto-*β transcripts are expressed throughout development, including larval stages. Using RT-PCR and qPCR analyses we also detected both transcripts in the third instar larval carcasses and estimated that the *neto-α* transcript was ~4 fold less abundant than *neto-β*. To examine if Neto-β is present at the NMJs, we generated Neto-β isoform specific antibodies (details in Materials and Methods and S2 Fig) and compared them with previously described Neto antibodies, raised against the extracellular CUB1 domain [12]. The Neto-β positive signals co-localized with the Neto-ex puncta at control and at *neto-β*-rescued NMJs, but not at *neto* null NMJs rescued with *neto-α* transgenes (Fig 1B). Importantly, muscle overexpression of either isoform could rescue the embryonic lethality and paralysis of *neto* null mutants. This suggests that the shared part of Neto, including the CUB1, CUB2, LDLa and the transmembrane pass, contains all the components essential for iGluRs clustering and NMJ development. Indeed, muscle expression of a Neto variant in which the intracellular domains were replaced by eGFP rescued the *neto* null paralyzed embryos to fertile adults (Fig 1B). The caveat of these rescue experiments is that Neto overexpression highly exceeds the endogenous levels and may obscure the contribution of individual isoforms. The function of the extracellular and transmembrane domains of *Drosophila* Neto will be examined elsewhere. Here we will focus on characterizing the new isoform, Neto-β, and its role during NMJ development.

We have previously demonstrated an essential role for Neto in the formation of iGluR clusters during synapse assembly and development. Likewise, Neto-β started to accumulate at the NMJ synapses at the onset of synaptogenesis (Fig 1C and 1D). In late embryo fillets, Neto-β antibodies labeled distinct synaptic puncta, which were also Neto-ex positive and co-localized with the iGluR synaptic signals (GluRIIA shown in Fig 1D). Neto-β signals remained at junctional locations throughout larval development and appeared to represent a significant fraction of the total Neto at the *Drosophila* NMJ (S2 Fig).

### Generation of *neto-β* isoform specific mutants

**Fig 2:**
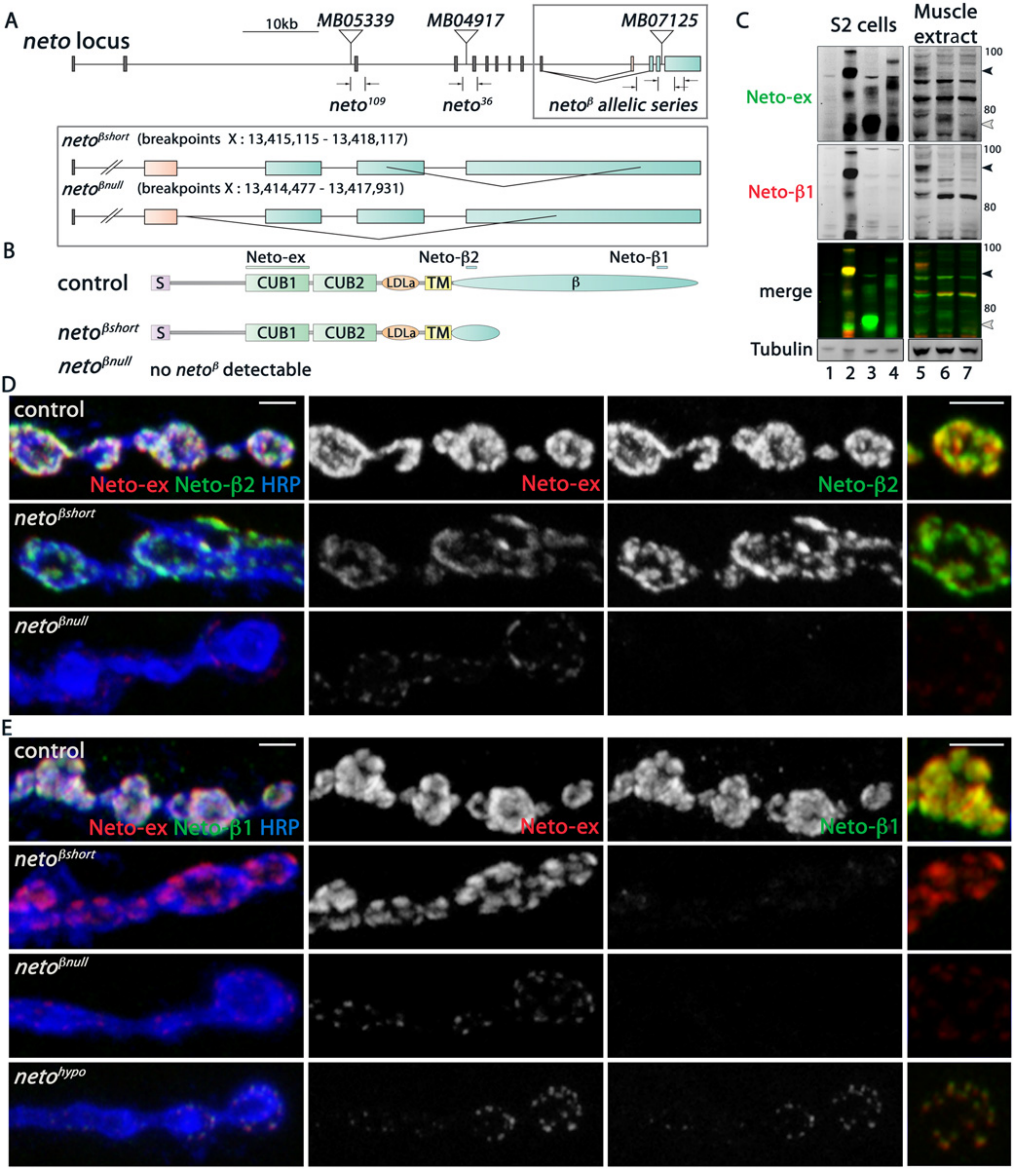
Generation and characterization of *neto-β* isoform specific alleles. (A) Schematics of the *Minos* transposomal elements and the small lesions corresponding to various *neto* alleles. *MB07125* was mobilized to generate precise excision control and isoform specific *neto-β* alleles, *neto* ^*βshort*^ (short cytoplasmic tail) and *neto* ^*βnull*^. The breakpoint coordinates are indicated. (B) Diagram of the predicted Neto-β proteins. The bars mark the antigens for Neto-ex, Neto-β1 and Neto-β2 antibodies. (C) Western blot analysis of lysates from S2 cells (left) and larval muscle (right). S2 cells were transfected with empty vector (lane 1), Neto-β (2), Neto-β^short^ (3), and Neto-α (4) expression constructs and the lysates were compared with muscle extracts from control (5), *neto* ^*βshort*^ (6), and *neto* ^*βnull*^ (7) third instar larvae. Full length (black arrow) and truncated (white arrow) Neto-β are indicated. No specific signal was detected in *neto* ^*βnull*^ animals. (D-E) Confocal images of boutons at NMJ4 of third instar larvae stained with Neto-ex (red) and with either Neto-β2 (D) or Neto-β1 (E) (green). As expected, Neto-ex signals were detected in control (precise excision) and *neto* alleles. *neto* ^*βshort*^ NMJs show Neto-β2 signals (D) but both *neto-β* alleles lack Neto-β1 synaptic signals (E). In contrast, Neto-β1 puncta are present at *neto* ^*hypo*^ NMJs. Scale bars: 2 μm.

To examine the role of Neto-β in synapse assembly and development we generated isoform specific mutants by imprecise excision of a transposable element Mi(ET1)Neto[MB07125] [25]. Several lines including precise excisions and small deletions were isolated and characterized by PCR amplification and sequencing (Fig 2A and Materials and Methods). The *neto* ^*203*^ allele is partly missing *neto-β* specific exons; *neto* ^*204*^ lacks the entire *neto-β* specific coding sequence and is likely a *neto-β* genetic null. No *neto-β* transcript was detectable in *neto* ^*204*^ animals, but in *neto* ^*203*^ larvae we found a truncated transcript, predicted to encode a short Neto-β variant (S1 Fig). For clarity we will refer to *neto* ^*203*^ as *neto* ^*βshort*^, *neto* ^*204*^ as *neto* ^*βnull*^, *neto* ^*36*^ as *neto* ^*null*^, and *neto* ^*109*^ as *neto* ^*hypo*^ [12]. A diagram of the *neto-β* alleles and predicted Neto-β proteins is shown in Fig 2A and 2B.

The predicted Neto-β variants were detected by Western analysis in larval muscle extracts (Fig 2C): a full-length Neto-β was detected in control, and a band of 65 kD, corresponding to the truncated Neto-β variant, was detected in *neto* ^*βshort*^ larval muscle. Interestingly, no band corresponding to Neto-α was detected suggesting that the endogenous Neto-α levels are very low, even in the absence of Neto-β. The Neto-β variants (full length and short) appeared expressed at equivalent levels relative to Tubulin. However, the synaptic Neto levels were decreased by 34% at *neto* ^*βshort*^ NMJs, suggesting that the cytoplasmic domain influences Neto distribution (Fig 2C and S3 Fig). The Neto levels were decreased by 70% at *neto* ^*βnull*^ NMJs, consistent with the estimated 4-fold difference in the transcript levels. Further purification of Neto-β polyclonal sera against the two antigenic peptides (β1 and β2) produced two pools of antibodies (Fig 2B and S1 Fig). As expected, only Neto-β2 antibody labeled the *neto* ^*βshort*^ synapses, whereas no Neto-β1 signals were found at *neto* ^*βshort*^ or *neto* ^*βnull*^ NMJs (Fig 2D and 2E). In contrast, clear Neto-β1 synaptic signals were detected at *neto* ^*hypo*^ NMJs. The *neto* ^*hypo*^ allele lacks the exon containing the translation initiation codon [12]. This lesion should affect both Neto isoforms.

We next examined the viability and behavior of *neto-β* mutants. Under optimal culturing conditions, 75% of *neto* ^*βnull*^ embryos and 90% of *neto* ^*βshort*^ developed into larval stages compared with control (n>200). In both cases, the third instar larvae were sluggish and exhibited a significant delay in rolling back when placed with dorsal side down. The *neto* ^*βshort*^ adult flies have no apparent defects, but the *neto* ^*βnull*^ homozygous have impaired climbing ability and are outcompeted by their heterozygous siblings. Further reduction to one copy of *neto*-*α*, such as in *neto* ^*βnull*^ */neto* ^*null*^ trans allelic combinations, induced 100% lethality in more than 600 progenies examined. This lethality was rescued by a duplication covering the *neto* gene, *Dp(1*:*3)DC270* [26], indicating that the molecular lesion is confined to the *neto* locus. In contrast, *neto* ^*βshort*^ */neto* ^*null*^ were viable indicating that one copy of *neto*-*α* and *neto-β* ^*short*^ could support adult viability.

### *neto-β* mutants have impaired NMJ physiology and morphology

**Fig 3:**
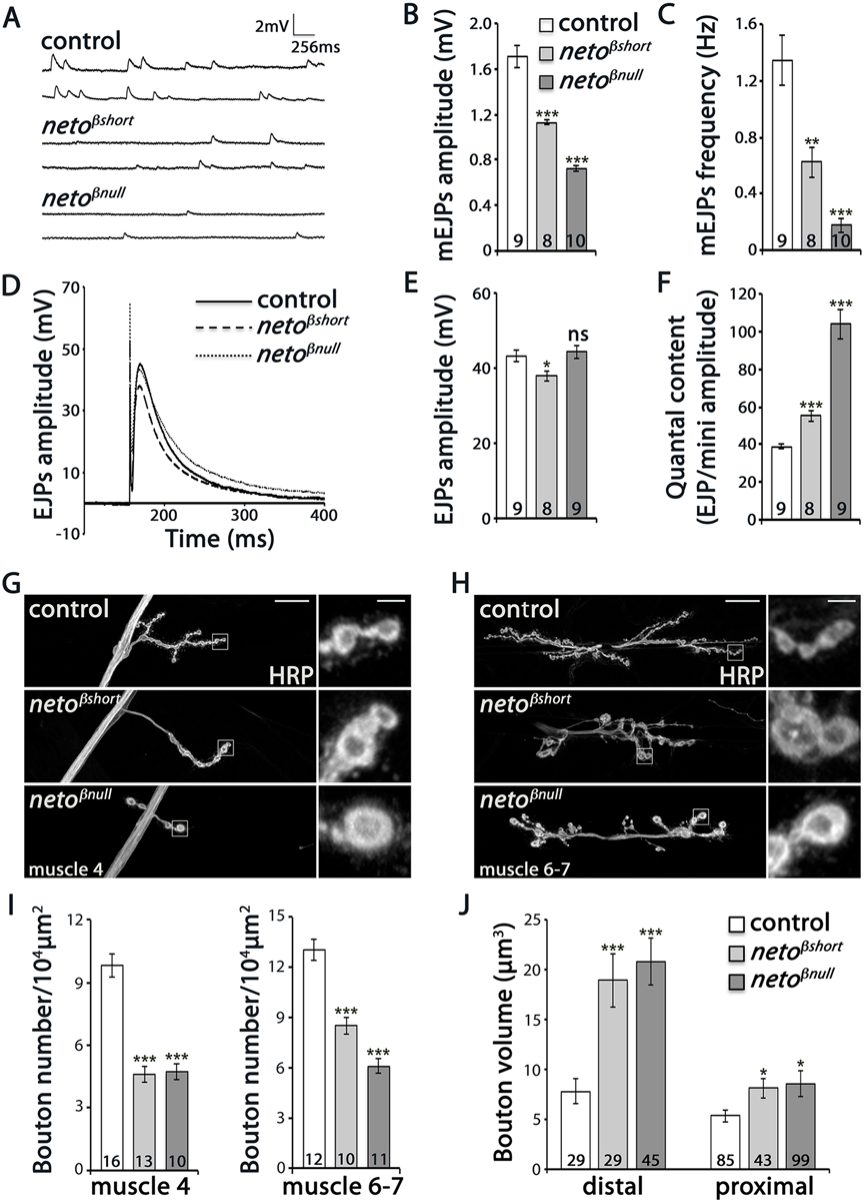
Physiology and morphology defects at *neto-β* mutant NMJs. (A–F) Electrophysiological recordings of control (precise excision) and *neto-β* alleles of third instar animals. Representative traces of mEJPs and EJPs at 0.8 mM Ca^2+^ are shown in (A) and (D), respectively, and the results are summarized in S1 Table. The number of NMJs examined is indicated in each bar. The mEJPs amplitude (B) and frequency (C) were reduced for both *neto-β* alleles. The EJPs amplitude was reduced in *neto* ^*βshort*^ compared to controls, but was normal at *neto* ^*βnull*^ NMJs (E). Both alleles had increased quantal content, indicating a presynaptic compensatory response (F). The muscle resting potential and the input resistance were not affected. (G-H) Representative confocal images at NMJ4 and NMJ6/7 (segment A3) in third instar larvae of indicated genotypes labeled with HRP. The *neto-β* mutant NMJs have significantly fewer and larger boutons relative to the muscle area (quantified in I-J). In particular, the volume of distal boutons increases 2.4 and respectively 2.6 fold at *neto* ^*βshort*^ and *neto* ^*βnull*^ NMJs. Error bars indicate SEM. ***; p<0.001, **; p<0.005, *; p<0.05, ns; p>0.05. Scale bars: 20 μm, 2 μm in details.

To test the functionality of NMJ synapses lacking an intact Neto-β isoform we recorded spontaneous miniature potentials (minis or mEJPs) and evoked excitatory junctional potentials (EJPs) from muscle 6 of third instar larvae. The mean frequency of mEJPs was decreased in both *neto-β* alleles compared to control (Fig 3A–3C and S1 Table). Mini amplitude or quantal size, the postsynaptic response to the fusion of a single vesicle, was also reduced in both *neto-β* alleles (to 62% of control in *neto* ^*βshort*^, and respectively 43% in *neto* ^*βnull*^). We found no significant change in the resting potential and input resistance in the *neto-β* mutant larvae.

The reduced mEJP frequency and amplitude at *neto-β* mutant NMJs were similar but less severe than at Neto- or iGluR-deprived NMJs [9,12]. But while Neto-deprived NMJs have severely reduced EJPs, the amplitude of EJPs was reduced by 12–14% compared to the control in *neto* ^*βshort*^ mutant larvae and was relatively normal at *neto* ^*βnull*^ NMJs (Fig 3D–3F and S1 Table). This suggests that *neto-β* mutant NMJs must compensate for the decreased postsynaptic sensitivity by a compensatory increase in quantal content, the number of vesicles released in response to each action potential. We found that quantal content, estimated as ratio of average EJP amplitude to the mEJP amplitude, was increased 1.4 fold in *neto* ^*βshort*^ larvae and 2.7 fold in *neto* ^*βnull*^ compared with control (Fig 3F). In contrast, the *neto* ^*hypo*^ or *neto* ^*RNAi*^ larvae show no presynaptic compensatory response to reduced quantal size [12,27].

These studies show that the loss of Neto-β significantly alters the number, type and/or density of postsynaptic iGluRs and elicits a homeostatic compensatory increase of presynaptic release. These changes resemble the *GluRIIA* loss-of-function phenotypes, and differ from Neto- or iGluR-deprived NMJs [7–12]. Thus, the defects observed in *neto-β* mutants do not seem to originate from a general decrease in Neto levels and suggest isoform specific Neto activities at the NMJ.

NMJ morphological analyses further emphasized these specific defects. We have previously shown that Neto-deprived NMJs have longer and fewer branches, with significantly reduced number of boutons [27]. In contrast, at similarly reduced Neto levels, the *neto* ^*βnull*^ NMJs were reduced in length and had shorter branches (Fig 3G and 3H). Compared with controls, *neto-β* mutant NMJs had fewer but larger type Ib boutons (Fig 3I and 3J). In particular, the distal boutons had an estimated 2.4 fold volume increase (*neto* ^*βshort*^: 18.9 μm^3^ ± 2.6, n = 29, *neto* ^*βnull*^: 20.8 μm^3^ ± 2.3, n = 45, and control: 7.8 μm^3^ ± 1.3, n = 29). Together these data indicate that perturbations of Neto-β have profound effects on the NMJ morphology and function that are different from a reduction in Neto levels.

### Loss of Neto-β affects PSD composition

**Fig 4:**
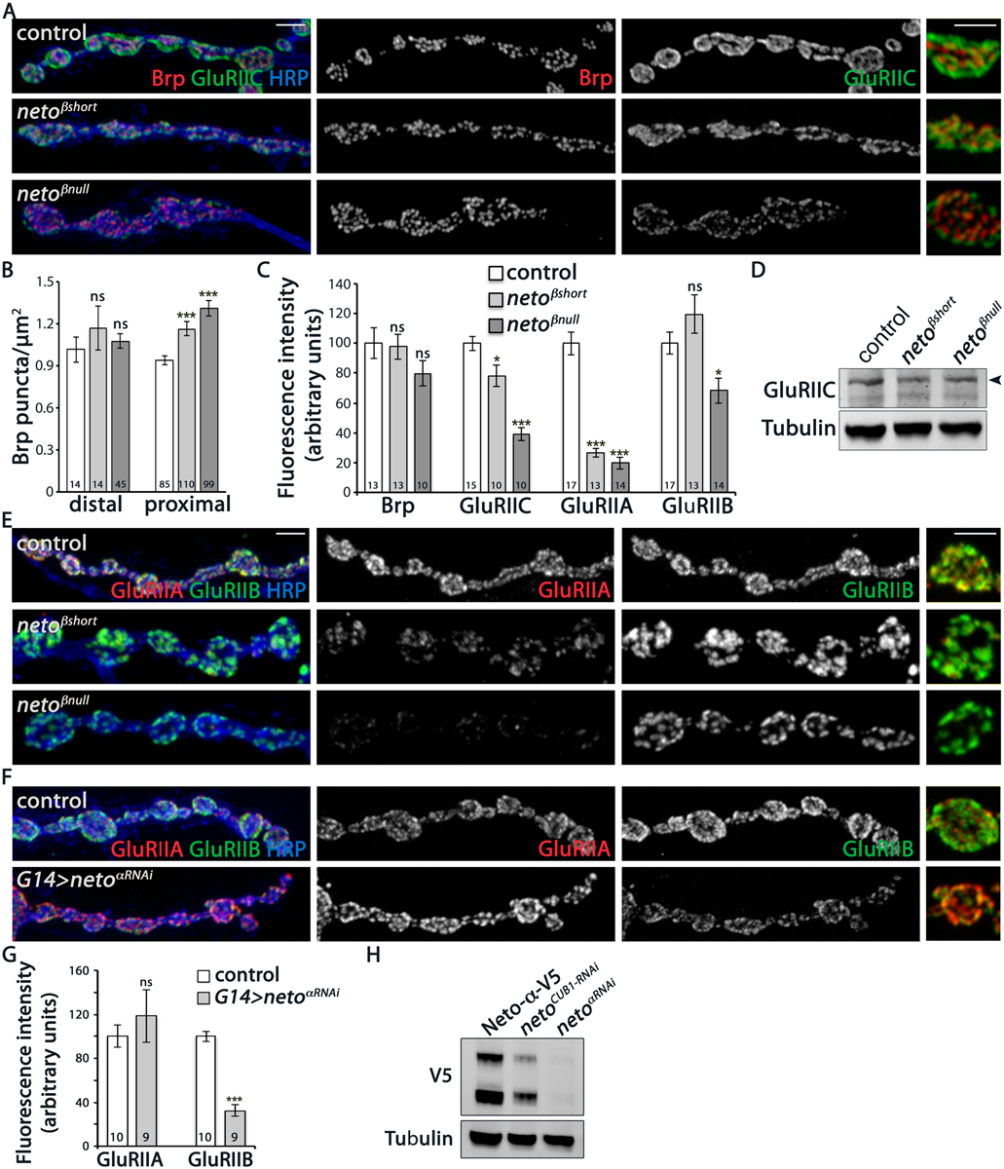
iGluRs synaptic accumulation is perturbed at *neto-β* mutant NMJs. (A) Confocal images of NMJ4 boutons in larvae of indicated genotypes labeled for Brp (red), GluRIIC (green), and HRP (blue) (quantified in B-C). *neto-β* mutant NMJs have increased number of synaptic contacts. The intensity of the presynaptic active zone marker Brp appears to be normal, but the GluRIIC synaptic signals are reduced at *neto-β* mutant NMJs compared with control (precise excision). (D) Western blot comparison of GluRIIC protein levels in muscle lysates from *neto-β* third instar larvae. Tubulin was used as a loading control. (E) Confocal images of NMJ4 boutons in larvae of indicated genotypes labeled for GluRIIA (red), GluRIIB (green), and HRP (blue). The synaptic accumulation of GluRIIA is severely reduced at *neto-β* mutant NMJs (quantified in C). In contrast, the GluRIIB synaptic signals are slightly increased at *neto* ^*βshort*^ NMJs and significantly reduced (by 31%) at *neto* ^*βnull*^ NMJs. (F) Confocal images of NMJ4 boutons in control and *neto-α* ^*RNAi*^ larvae labeled for GluRIIA (red), GluRIIB (green), and HRP (blue). The GluRIIB signals, but not GluRIIA are reduced at Neto-α-depleted NMJs (quantified in G). (H) Western blot analysis of larval muscle extracts from *neto* ^*null*^ mutants rescued by V5-tagged Neto- and carried through RNAi-mediated knockdown as indicated. The *neto-* ^RNAi^ appears to be more effective than the *CUB1* ^*RNAi*^ in knocking down V5-tagged Neto- relative to the Tubulin control. Error bars indicate SEM. ***; p<0.001, **; p<0.005, *; p<0.05, ns; p>0.05. Scale bars: 2 μm.

At the *Drosophila* NMJ, Neto activities are essential for trafficking and clustering of iGluRs at synaptic sites. To assess the contribution of Neto-β to these functions we examined the iGluRs distribution at *neto-β* mutant NMJs. For all the quantitative analyses below control/*neto-β* mutant sets (5–10 larvae/genotype) were processed together and imaged under the same conditions to capture and compare the synaptic signals. At the fly NMJ synapses, the sites of neurotransmitter release are marked by presynaptic specializations called T-bars, where Bruchpilot (Brp), the fly homolog of the vertebrate active zone protein ELKS, accumulates [28]. Opposite to the T-bars, the iGluRs are concentrated and stabilized at synaptic sites by a myriad of PSD-associated proteins. We found that *neto-β* mutant boutons contained more synaptic contacts, as visualized by Brp/GluRIIC juxtaposing puncta, and their density was increased at proximal boutons but not at the distal ones (Fig 4A and 4B). This increase in synaptic density is consistent with the compensatory response observed at *neto-β* mutant NMJs (Fig 3F). The intensity of Brp signals was largely constant, but the GluRIIC levels were diminished to 78% from control at *neto* ^*βshort*^ NMJs (n = 10) and to 39% at *neto* ^*βnull*^ NMJs (n = 10) (Fig 4A and 4C). The reduction of GluRIIC synaptic signals at *neto-β* mutant NMJs occurred without a change in the GluRIIC net protein indicating a defect in the synaptic localization of the receptors (Fig 4D). Importantly, the GluRIIC synaptic signals paralleled the levels of synaptic Neto observed at these NMJs (Fig 2D and 2E, S3A Fig), as Neto is limiting for receptors localization.

*Drosophila* NMJ utilizes two types of iGluRs, type-A and type-B, that differ in their subunits composition and properties [13]. The single channel current amplitude of both receptor types is identical, but the type-B channel desensitizes nearly ten times faster, thus the relative ratio of synaptic type-A/type-B receptors is a key determinant of quantal size [8]. Also, reduced levels of postsynaptic type-A receptors trigger a robust presynaptic compensatory response [7]. The physiological similarities between the *GluRIIA* and *neto-β* mutant NMJs suggest that Neto-β may primarily influence the synaptic accumulation of type-A receptors. We tested this possibility by examining the relative intensity of GluRIIA and GluRIIB signals at *neto-β* mutant NMJs. The *neto* ^*βshort*^ larvae had severely reduced GluRIIA synaptic levels (to 26% from control, n = 13), accompanied by no significant change in the GluRIIB levels (Fig 4C and 4E). The *neto* ^*βnull*^ NMJs showed an even stronger reduction in the GluRIIA synaptic levels (to 18.5% from control, n = 14) and also decreased GluRIIB levels (Fig 4C and 4E). In contrast, the *neto* ^*hypo*^ NMJs showed uniformly reduced levels for all iGluR subunits examined (S3 Fig). The drastic decrease of GluRIIA synaptic signals at *neto-β* mutant NMJs exceeded the reduction of GluRIIC and Neto synaptic levels observed at these NMJs, indicating a key role for the Neto-β isoform in regulating the GluRIIA synaptic abundance.

How does Neto-β regulate the selective accumulation of type-A receptors at synaptic sites? Neto-β may preferentially recruit the type-A receptors and/or influence their synaptic stabilization. If Neto isoforms promote the synaptic accumulation of selective iGluRs, and Neto-β influences the type-A receptors, the inference is that Neto-α should preferentially impact the distribution of type-B receptors. In support for this possibility, in RNAi knockdown experiments we found that Neto-α-deprived NMJs showed a dramatic loss of GluRIIB synaptic signals (by 68%) and a modest, variable increase of synaptic GluRIIA (by 18%) (Fig 4F and 4G). The efficiency of the RNAi-mediated knockdown was verified by Western blot analysis of muscle extracts from *neto* ^*null*^ larvae rescued with a V5-tagged *neto-α* transgene (Fig 4H and [12]). Lack of Neto-α-specific reagents precluded us from expanding these experiments. Nonetheless, these results raise the possibility that Neto isoforms utilize their distinct cytoplasmic domains to modulate the synaptic abundance of specific receptor subtypes. In the case of Neto-β, the last 260 C-terminal cytoplasmic residues appear to be critical for the synaptic accumulation of type-A receptors.

### Neto-β recruits postsynaptic components and organizes postsynaptic structures

In *Drosophila*, several postsynaptic and perisynaptic proteins influencing the synaptic abundance of type-A and type-B receptors have been reported [15,16,18]. For example, PAK, a PSD component that co-localizes with the GluRIIA subunit, was shown to promote accumulation of type-A receptors at synaptic sites [16,17,29]. PAK also influences the size of the SSR, a stack of membrane folds that underlines the postsynaptic cell membrane of the type Ib boutons [16,17].

**Fig 5:**
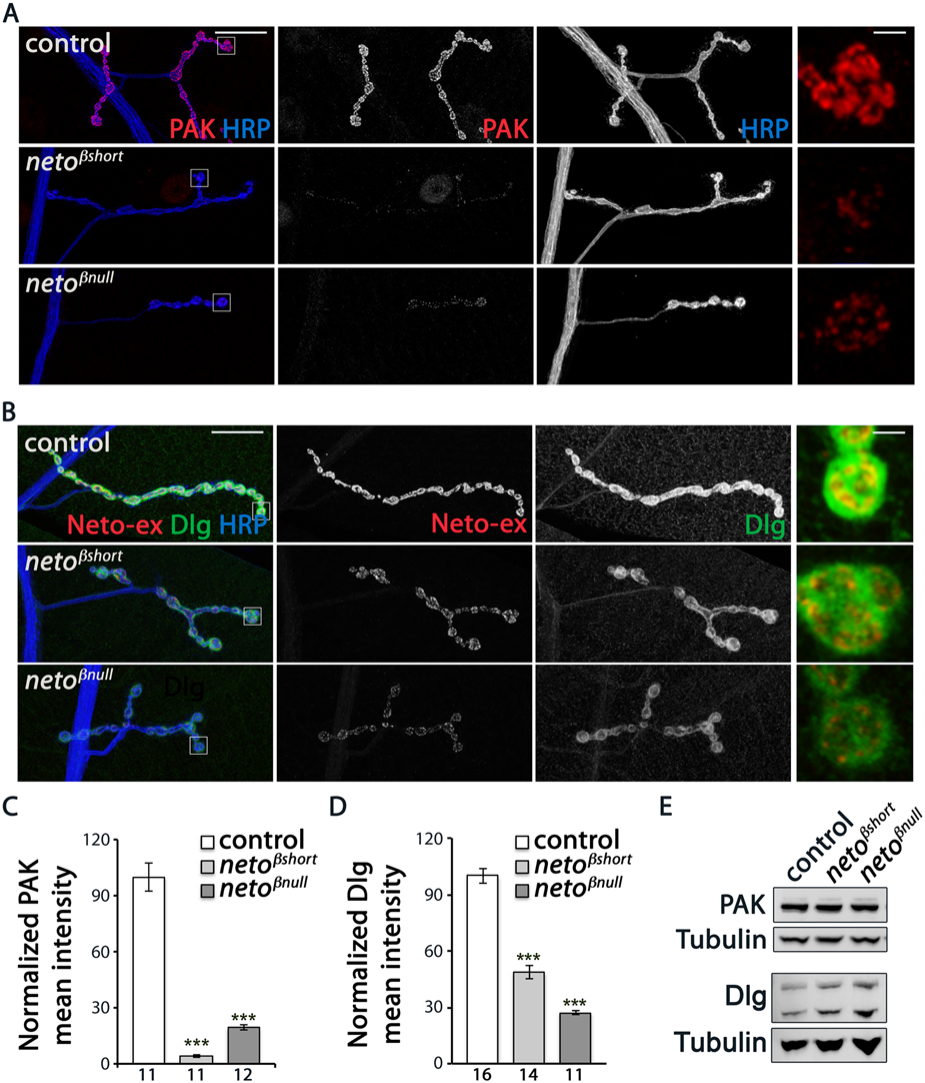
*neto-β* mutants have reduced postsynaptic components. (A–B) Representative confocal images at NMJ4 (segment A3) in third instar larvae of indicated genotypes labeled for HRP (blue) and PAK (red) (A) or Neto-ex (red) and Dlg (green) (B). The *neto-β* mutant NMJs have drastically reduced levels of synaptic PAK and significantly diminished Dlg accumulation (quantified in C-D), albeit the protein levels are normal in larval muscles as indicated by Western blot analysis (E). Tubulin was used as a loading control. Error bars indicate SEM. ***; p<0.001. Scale bars: 20 μm, 2 μm in details.

We found that PAK signals were severely reduced at *neto-β* mutant NMJs (Fig 5A). PAK synaptic levels dropped to 4% from control in *neto* ^*βshort*^ larvae (n = 11) and to 19% in *neto* ^*βnull*^ (n = 12). This drastic decrease exceeded the reduction in PAK signals observed at *neto* ^*hypo*^ NMJs, suggesting that the low level of Neto-β at *neto* ^*hypo*^ NMJs could partly mediate PAK stabilization at PSDs (Fig 2E and [12]). A similar, albeit less pronounced effect was observed for Dlg (Fig 5B–5D). The Dlg junctional signals were reduced to 48% from control in *neto* ^*βshort*^ third instar larvae (n = 14) and to 27% from control in *neto* ^*βnull*^ (n = 11). Importantly, the levels of PAK and Dlg net proteins were not changed in extracts from *neto-β* larval muscles (Fig 5E). Also, the accumulation of PAK and Dlg at junctional locations was completely restored in *neto-β* mutants by a duplication covering the *neto* locus (S4 Fig), indicating that the observed phenotypes are *neto* specific. In contrast, cysteine string protein (CSP), which labels clusters of synaptic vesicles in the vicinity of presynaptic membranes [30], and α-Spectrin, which labels the presynaptic axon and surrounds the bouton postsynaptically [31] appeared normal at *neto-β* mutant NMJs (S5 Fig).

**Fig 6:**
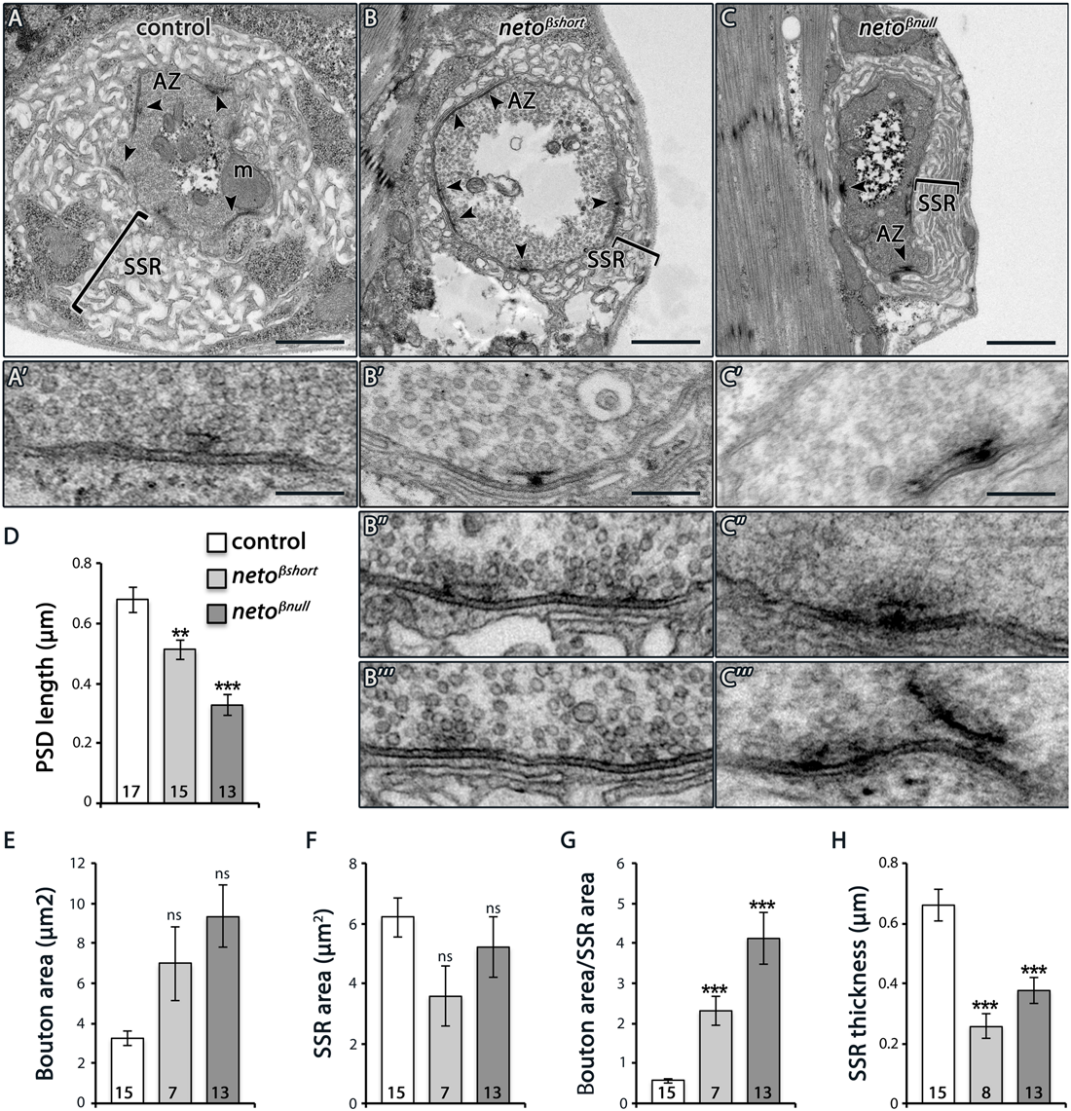
Ultrastructure defects at *neto-β* mutant boutons. (A–C) Electron micrographs of type Ib boutons in third instar larvae of indicated genotypes. The upper panels show entire boutons; the active zones (AZ, arrows), mitochondria (m), and subsynaptic reticulum (SSR, brackets) are indicated. The *neto-β* mutant boutons have numerous synaptic contacts, but their active zones often have abnormal T-bar structures (B’-B’”, C’-C’”), including closely spaced, distorted, fused, and floating T-bars. The PSDs length, quantified in serial section (D), is significantly reduced at *neto-β* mutant boutons. The boutons appear enlarged and the SSR area and thickness diminished in both *neto-β* mutants (quantified in E-H). Error bars indicate SEM. ***; p<0.001, **; p<0.005. Scale bars: 1 μm, 200 nm in details.

Under electron microscopy, control synapses are marked by presynaptic specializations called T-bars and electron-dense membranes, corresponding to PSDs (Fig 6A–6C). At *neto-β* mutant boutons, serial sections revealed that the PSDs were significantly reduced in size but increased in number (Fig 6B–6D and Fig 4). The diminished PSD length (up to 2 fold in *neto* ^*βnull*^ mutants) matched the observed reduction of postsynaptic receptor fields (Fig 4). A thick stack of SSR membranes surrounds the control type Ib boutons. In contrast, the enlarged *neto-β* mutant boutons had diminished or even absent SSR structures, consistent with the reduced Dlg levels observed (Fig 6E–6H). In addition, *neto-β* mutants appeared to have misshaped T-bar structures, including double T-bar structures (Fig 6B and 6C, details). GluRIIA deprivation induces excessive recruitment of Brp at the active zones which sometimes results in active zone profiles with two or more T-bars [32]. Likewise, the excessive T-bar structures observed primarily in *neto* ^*βnull*^ mutant boutons may correlate to a homeostatic compensatory response triggered by the GluRIIA depletion (Figs 3F and 4B and 4E).

### Neto-β, but not Neto-α, is required for PAK accumulation at PSDs

**Fig 7:**
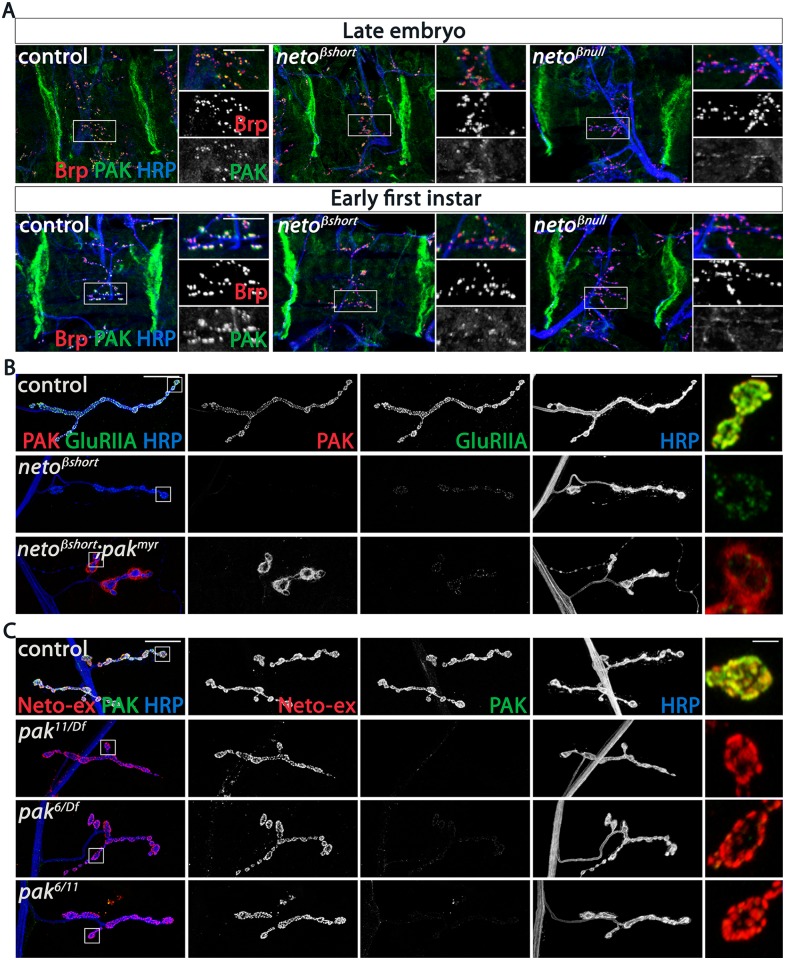
PAK synaptic recruitment is impaired at *neto-β* mutant NMJs. (A) Representative confocal images of ventral muscle fields in late embryos (21 hours after egg laying) and early first instar larvae (2 hours after hatching) of indicated genotypes labeled for Brp (red), PAK (green), and HRP (blue). The synaptic PAK signals are weak at *neto-β* mutant NMJs, but normal at the muscle attachment sites. (B) Confocal images of NMJ4 in third instar larvae of indicated genotypes labeled for PAK (red), GluRIIA (green) and HRP (blue). Muscle expression of a constitutively membrane bound form of PAK (*G14> pak* ^*myr*^) does not rescue the GluRIIA synaptic abundance at *neto* ^*βshort*^ NMJs compared to control (*G14 /+*). PAK signals remain diffuse and localize perisynaptically in the absence of an intact Neto-β (see bouton details). (C) Confocal images of NMJ4 in control (*w* ^*1118*^) and various *pak* heteroallelic third instar larvae labeled for Neto-ex (red), PAK (green) and HRP (blue). Lack of PAK does not impact the synaptic distribution of Neto-β. Scale bars: (A) 10 μm; (B-C) 20 μm, 2 μm in details.

Since the SSR folds are reduced in *neto-β* mutants, it is possible that the reduced PAK and Dlg synaptic levels are indirectly caused by the lack of proper SSR structures. To test this possibility, we examined late embryos and first instar larvae, before the SSR develops [33]. If the SSR controls the synaptic accumulation of PAK, then PAK levels should be normal at *neto-β* mutant NMJs during the early stages of development. However, we found that PAK accumulated normally at the muscle attachment sites in control and *neto-β* mutant animals, but was severely diminished at *neto-β* mutant NMJs (Fig 7A). Thus, loss of synaptic PAK is not secondary to the loss of SSR and appears to be directly caused by impairments in Neto-β.

If Neto-β recruits PAK at the cell membrane, then loss of synaptic PAK in *neto-β* mutants should be rescued by muscle expression of PAK^myr^, a membrane-tethered PAK variant [34]. We found that muscle overexpression of PAK^myr^ in *neto-β* mutants induced accumulation of PAK signals at perisynaptic but not synaptic sites, and could not rescue the GluRIIA synaptic accumulation (*neto* ^*βshort*^ shown in Fig 7B). This indicates that Neto-β controls the recruitment of PAK at synaptic locations. Loss of PAK did not affect the synaptic recruitment of Neto-β: while PAK signals were lost at *pak* null NMJs (*pak* ^*11/Df*^) or in mutants that fail to localize at cell peripheries (*pak* ^*6/Df*^or *pak* ^*6/11*^) [35], the synaptic Neto signals remained unaffected, irrespective of the *pak* genetic manipulations (Fig 7C). Also, the levels of GluRIIA were 4-fold reduced in *neto-β* mutants (Fig 4), but only 2-fold in *pak* mutants [16], suggesting that Neto-β has additional, PAK-independent functions in the synaptic recruitment/stabilization of GluRIIA. Together these data indicate that Neto-β is required for the synaptic accumulation of PAK. In flies and mammals, PAK membrane localization is controlled by Pix, a Rho-type guanine nucleotide exchange factor [16,36]. Mutations in *dpix* led to impairments in synaptic accumulation of PAK, GluRIIA, Dlg, and other postsynaptic components. Lack of Pix antibodies precluded us to test whether Neto-β recruits dPix at PSDs. Nonetheless, while the PAK synaptic signals were completely lost in *dpix* mutants, Neto signals accumulated at these mutant synapses albeit they appeared somewhat reduced, likely due to defects in the synapse organization (S6 Fig). Thus, dPix is required for PAK but not for Neto recruitment of at synaptic sites.

**Fig 8:**
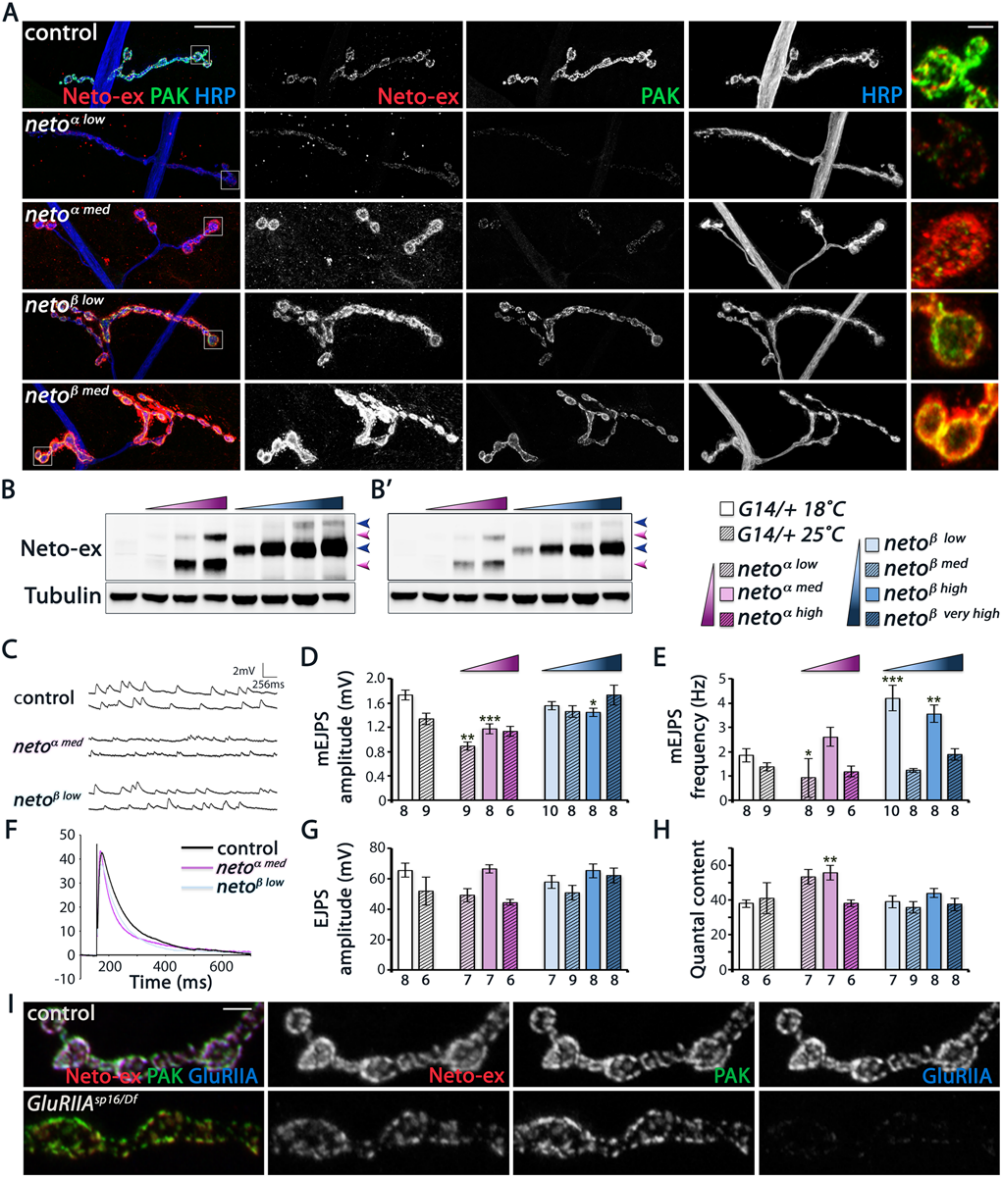
Neto-β, but not Neto-α, restores PAK recruitment and mEJP amplitude at *neto* ^*null*^ NMJs. (A) Confocal images of larval NMJ4 labeled for Neto-ex (red), PAK (green) and HRP (blue). Neto-β, but not Neto-α, restores PAK synaptic accumulation over a large range of concentrations tested. (B) Western blot comparison of Neto levels in muscle extracts from control (first lane), and *neto* ^*null*^ larvae rescued with *neto-α* transgenes (low, medium, and high expression) (magenta gradient), or *neto-β* transgenes (low, medium, high, and very high expression)(blue gradient). Arrows indicate unprocessed and processed Neto-α (magenta) and Neto-β (blue). (B’)—low exposure. Tubulin was used as a loading control. (C-H) Electrophysiological recordings of *neto* ^*null*^ NMJs rescued by various levels of Neto-α or Neto-β. Data are reported relative to controls matched by rearing at 18°C (empty bars) or 25°C (hatched bars). Representative traces of mEJPs and EJPs are shown in (C) and (F), respectively. The mEJPs amplitude is reduced at NMJs rescued by low and medium levels of Neto-α (D). The mEJPs frequency appears less dependent on Neto levels/isoforms, but is significantly increased in larvae reared at 18°C (E). The EJPs amplitudes are largely normal (G), likely due to subtle, but significant increases in quantal content at Neto-α-rescued NMJs (H). (I) Confocal images of NMJ4 boutons (segment A3) in control (*w* ^*1118*^) and *GluRIIA* ^*SP16/Df*^ (*GluRIIA* ^*SP16*^/ *Df(2L)cl* ^*h4*^) third instar larvae labeled for Neto-ex (red), PAK (green), and GluRIIA (blue). PAK is normally present at *GluRIIA* mutant synapses, indicating that the synaptic recruitment PAK does not depend on GluRIIA. Genotypes: control (*G14-Gal4/+*); *neto* ^*α low*^ (*neto* ^*36*^ */Y*; *G14-Gal4/UAS-neto-A9*), reared at 25°C); *neto* ^*α med*^ (*neto* ^*36*^ */Y*; *G14-Gal4/UAS-neto-A3*, 18°C); *neto* ^*α high*^ (*neto* ^*36*^ */Y*; *G14-Gal4/UAS-neto-A3*, 25°C); *neto* ^*β low*^ (*neto* ^*36*^, *UAS-neto-B6/Y*; *G14-Gal4/+*, 18°C); *neto* ^*β med*^ (*neto* ^*36*^, *UAS-neto-B6*/Y; *G14-Gal4/+*, 25°C); *neto* ^*β high*^ (*neto* ^*36*^ */Y*; *G14-Gal4/UAS-neto-B3*, 18°C); *neto* ^*β very high*^ (*neto* ^*36*^ */Y*; *G14-Gal4/UAS-neto-B3*, 25°C). Error bars indicate SEM. ***; p<0.001, **; p<0.005, *; p<0.05, ns; p>0.05. Scale bars: 20 μm, 2 μm in details and (I).

We have previously demonstrated that the net levels of Neto are critical for synapse assembly and NMJ development [12,14]. In particular, Neto depletion has profound consequences for synapse assembly and function. The levels of synaptic Neto are reduced in *neto-β* mutants compared with controls (Fig 2 and S3 Fig) raising the possibility that the phenotypes observed in *neto-β* allelic series could be partly due to reduced Neto levels. To distinguish between isoform specific and suboptimal Neto phenotypes we manipulated the muscle expression of Neto-α and Neto-β isoforms and examined their effects on the development and function of NMJ. Similar to *neto-α*, we found that muscle expression of *neto-β* transgenes rescued the viability and NMJ development of *neto* ^*null*^ mutants and induced gain-of-function phenotypes in a concentration-dependent manner (Fig 8A and [27]). Low to moderate levels of Neto-β induced formation of NMJs with relatively normal morphology and clearly defined synaptic Neto clusters, resembling the *neto* mutants rescued with low and moderate levels of Neto-α. Excess Neto-β appeared detrimental to the NMJ development and to the overall growth and viability of rescued animals (Fig 8A).

Intriguingly, Neto-β but not Neto-α enabled the stabilization of PAK at PSDs, over a wide range of concentrations tested (Fig 8A). Furthermore, increasing the levels of Neto- α in *neto-β* mutants could not restore the PAK synaptic accumulation, while introducing Neto-β effectively rescued this defect. This result cannot be explained by a difference between Neto- α and Neto-β cellular distribution, since both isoforms appear to concentrate at the NMJ. We confirmed that Neto-α and Neto-β synaptic levels correlate with the levels of Neto protein in the larval muscle, as detected by Western analysis (Fig 8B). Thus, both Neto isoforms can traffic and accumulate at synaptic locations where they mediate synapse assembly. However, only Neto-β isoform can enable PAK accumulation at PSDs.

Since PAK contributes to synaptic stabilization of GluRIIA, Neto-α-rescued NMJs are expected to exhibit a reduction in the synaptic GluRIIA signals and thus a reduction in quantal size. Indeed, we found that *neto* ^*null*^ mutants rescued with low/moderate levels of Neto-α had significantly reduced mini amplitudes and reduced GluRIIA/GluRIIB ratio (Fig 8C and 8D, S7 Fig). In contrast, *neto* ^*null*^ NMJs rescued with *neto-β* transgenes had relatively normal mini amplitudes, indicating normal GluRIIA/GluRIIB ratio at these synapses. The mini frequency was largely normal, except for the *neto-β* transgenes which showed increased mini frequency when reared at 18°C (Fig 8E). Interestingly, the evoked potentials were in the normal range in all Neto-α and Neto-β-rescued larvae tested, likely due to compensatory presynaptic response (Fig 8F–8H). These results indicate that Neto-α-rescued NMJs have deficits in the synaptic accumulation of type-A receptors. Such defects are partly obscured by excess Neto-α, likely because the conserved domains of Neto confer high iGluRs “clustering capacity” in these rescue experiments. But under normal condition, Neto is a low abundance protein and a limiting factor for iGluRs clustering in the muscle. Furthermore, Neto-β appears to be the predominant isoform at the *Drosophila* NMJ. Together our findings demonstrate that Neto-β, the major Neto isoform at the *Drosophila* NMJ, controls the subtype composition of iGluRs partly by regulating the recruitment of the PSD-associated kinase PAK.

## Discussion

At the *Drosophila* NMJ, Neto enables iGluRs clustering at synaptic sites and promotes postsynaptic differentiation. Here we show that Neto-β, the major Neto isoform at the fly NMJ, plays a crucial role in controlling the distribution of specific iGluR subtypes at individual synapses. Similar to other glutamatergic synapses, the subunit composition determines the activity and plasticity of the fly NMJ. Our data are consistent with a model whereby Neto-β, via its conserved domains, fulfills a significant part of Neto-dependent iGluRs clustering activities during synapse assembly. At the same time, Neto-β engages in intracellular interactions that regulate iGluR subtypes distribution by preferentially recruiting and/or stabilizing type-A receptors. In this model, Neto-β could directly associate with the GluRIIA-containing complexes and/or regulate the synaptic abundance of type-A receptors indirectly, by recruiting PSD components such as PAK. Thus, Neto-β employs multiple strategies to control which flavor of iGluR will be at the synapses and to modulate PSD composition and postsynaptic organization.

### Neto-mediated intracellular interactions shape postsynaptic composition

Neto proteins have been initially characterized as auxiliary subunits that modulate the function of kainate (KA) and NMDA receptors [22,23]. In vertebrates, Neto1 and Neto2 directly interact with KAR subunits and increase channel function by modulating gating properties [23,37,38]. Since loss of KAR currents in mice lacking Neto1 and/or Neto2 exceed a reduction that could be attributed to alterations of channel gating, an additional role for Neto proteins in synaptic targeting of receptors has been proposed. The role for vertebrate Neto proteins in KAR membrane and/or synaptic targeting remains controversial and appears to be cell type-, receptor subunit-, and Neto isoform-dependent [23,39,40]. Furthermore, the *C*. *elegans* Neto has a very small intracellular domain (24 amino acids beyond the conserved domains) [24]. This implies that 1) Neto without an intracellular domain constitutes the minimal conserved functional moiety, and 2) the divergent intracellular domains of Neto proteins may fulfill tissue and/or synapse specific modulatory functions. Indeed, Neto2 bears a class II PDZ binding motif that binds to the scaffold protein GRIP and appears to mediate KARs stabilization at selective synapses [41].

In flies, Neto is an essential protein that plays active roles in synapse assembly and in the formation and maintenance of postsynaptic structures at the NMJ. The *Drosophila* Neto isoforms do not have PDZ binding motifs, but they use at least two different mechanisms to regulate the synaptic accumulation and subunit composition of iGluRs. First, Neto participates in extracellular interactions that enable formation of iGluR/Neto synaptic complexes; formation of stable aggregates is presumably prevented by the inhibitory prodomain of Neto [27]. Second, the two Neto isoforms appear to facilitate the selective recruitment and/or stabilization of specific iGluR subtypes. We speculate that Neto-β may selectively associate with and recruit type-A receptors, perhaps by engaging the C-terminal domain of GluRIIA, which is critical for the synaptic stabilization of these receptors [42,43]. Aside from a possible role in the selective recruitment of iGluR subtypes, Neto-β participates in intracellular interactions that facilitate the recruitment of PAK at PSDs; in turn, PAK signals through two independent, genetically separable pathways (a) to modulate the GluRIIA synaptic abundance and (b) to facilitate formation of SSR [17].

Whether Neto-β recruits PAK directly or via a larger protein complex remains to be determined. Neto-β contains an SH3 domain that may bind to the proline-rich SH3 binding domain of PAK. However, in tissue culture experiments, we failed to detect a direct interaction between PAK and Neto-β (full-length or intracellular domain). PAK synaptic accumulation is completely abolished at NMJ with mutations in dPix, although not all *dpix* defects are mediated through PAK [16]. Conversely, PAK together with Dreadlocks (Dock) controls the synaptic abundance of GluRIIA, while PAK and dPix regulate the Dlg distribution [17]. The reduction of GluRIIA and Dlg synaptic abundance observed at *neto-β* mutant NMJs suggests that Neto-β may interact with both dPix and Dock and enable both PAK activities. In addition, Neto-β may stabilize postsynaptic type-A receptors by enhancing their binding to Coracle, which anchors GluRIIA to the postsynaptic actin cytoskeleton [15].

Importantly, this study connects the complex regulatory networks that modulate the PSD composition to the Neto/iGluR clusters themselves. The Neto-β cytoplasmic domain is rich in putative protein interaction motifs (S1 Fig), and may function as a scaffold platform to mediate multiple protein interactions that act synergistically during synapse development and homeostasis. Loss of Neto-β-mediated intracellular interactions at *neto* ^*βshort*^ NMJs reduced the GluRIIA synaptic abundance, but did not affect the GluRIIB synaptic signals (Fig 4E). It is unlikely that the remaining cytoplasmic part of Neto-β facilitates the GluRIIB synaptic accumulation at these NMJs at the expense of GluRIIA and PAK. Instead, we favor a model whereby the synaptic stabilization of GluRIIA requires a Neto-β-dependent intracellular network. Disruption of this network diminishes GluRIIA and increases GluRIIB synaptic abundance, pending the availability of limiting subunits, GluRIIC-E and Neto. Conversely, the presence of this network ensures that at least some type-A receptors are stabilized at synaptic sites, even at Neto-deprived synapses, such as in *neto* ^*hypo*^ larvae [12]. Assembly of this network does not require GluRIIA since both Neto-β and PAK accumulated normally at *GluRIIA* mutant NMJs (Fig 8I). Furthermore, in the absence of Neto-β the synaptic abundance of GluRIIA can be partly restored by excess Neto-α or a delta-intracellular Neto variant, suggesting that excess iGluRs “clustering capacity” overrides the cellular signals that shape PSD composition [27]. What intracellular domain(s) of Neto bind to and how they are modified by post-translational modifications will be critical questions to understand how postsynaptic composition is regulated during development and homeostasis.

### Expanding the repertoire of Neto functions at glutamatergic synapses

The discovery of *Drosophila* Neto isoforms with alternative cytoplasmic domains and isoform specific activities expands the repertoire of Neto-mediated functions at glutamatergic synapses. On one hand, all Neto proteins share the highly conserved CUB1, -2, LDLa and transmembrane domains that have been implicated in engaging and modulating the receptors, the central function of Neto proteins [22,23,44]. In flies this conserved part is both required and sufficient for iGluRs clustering and NMJ development. In *C*. *elegans* the entire Neto appears to be reduced to this minimal functional unit [24]. The only exception is a retina-specific CUB1-only Neto1 isoform with unknown function [45]. In contrast, the cytoplasmic domains are highly divergent among Neto proteins. This diversity might have evolved to facilitate intracellular, context specific function for Neto proteins, such as the need to couple the iGluR complexes to neuron or muscle specific scaffolds in various phyla. Alternatively, by engaging in different intracellular interactions, via distinct cytoplasmic domains, different Neto isoforms may undergo differential targeting and/or retention at the synapses and thus acquire isoform-specific distributions and functions within the same cell.

Phylogenetic analyses indicate that the intracellular domains of Neto are rapidly evolving in insects. Blocks of high conservations could be clearly found in the genome of short band insect *Tribolium castaneum* (*Coleoptera*) or in *Apis mellifera* (*Hymenoptera*). However, most insects outside *Diptera* appear to have only one Neto isoform, more related to Neto-β. In fact, the only Neto-α isoform outside *Drosophila* was found in *Musca domestica* (unplaced genomic scaffold NCBI Reference Sequence: XM_005187241.1). Other *neto* loci, from *Hydra* to vertebrates, appear to encode Neto proteins with unique and highly divergent intracellular domains. An extreme example is the *C*. *elegans* Neto/Sol-2, with a very short cytoplasmic tail, which requires additional auxiliary subunits, Sol-1 and Stargazin, to control the function of glutamate receptors [46,47]. Neto proteins appear to utilize their intracellular domains to connect to the signaling networks that regulate the distribution and subunit composition for iGluRs. Such cellular signals converge onto and are integrated by the intracellular domains of the receptors and/or by various auxiliary subunits associated with the iGluR complexes [1,6].

Neto proteins modulate the gating behavior of KAR but also play crucial roles in the synaptic recruitment of glutamate receptors in vivo [12,40,48]. At the fly NMJ, Neto enables iGluRs synaptic clustering and initiates synapse assembly. In addition, the intracellular domain of Neto-β recruits PSD components and triggers a cascade of events that organize postsynaptic structures and shape the composition of postsynaptic fields. The cytoplasmic domains of Neto proteins emerge as versatile signaling hubs and organizing platforms that directly control the iGluRs subunit composition and augment the previously known Neto functions in modulation of glutamatergic synapses.

## Materials and Methods

### Fly stocks

To generate *neto-β* alleles, the Minos transposomal element Mi(ET1)Neto[MB07125] was mobilized with Minos transposase [25]. Several lines with precise excisions and imprecise excisions/small deletions were isolated and characterized by PCR amplification over the deficiencies and DNA sequencing. The genomic fragments removed in various *neto* alleles were as follows: *neto* ^*203*^ X: 13,415,115–13,418,117, including part of the exon 13 and 14 of the predicted *neto* gene, and *neto* ^*204*^ X: 13,414,477–13,417,931, containing exons 12, 13 and 14. The *UAS-neto-β* lines (B lines) were generated by insertion of the *neto-β* cDNA (from RE42119) in pUAST vector and germline transformation (BestGene, Inc.). Similarly, for the *UAS-netoΔintra* (the H4 line) the Neto coding sequence M^1^-R^471^ followed by a short linker (DVPALE) was placed in frame with the eGFP and cloned in pUAST. The *neto-α* ^*RNAi*^ lines were generated by insertion of a *neto-α* specific PCR fragment in pUAST-R57 followed by germline transformation. The PCR primers utilized were: RNAi-F (5′-AAGGCCTACATGGCCGGACCGGCGAACAAATGGAGGAAGACG-3′) and RNAi-Rev (5′-AATCTAGAGGTACCTGATTTTGTGCAGGAACTTGAGG-3′).

Other fly stocks used in this study were as follows: *neto* ^*36*^, *neto* ^*109*^, *and UAS-neto-α -V5 [12]; UAS-neto-α* (A lines) [27]; *neto(CUB1)* ^*RNAi*^ [14]; *Dp(1*:*3)DC270* [26]; *pak* ^*6*^, *pak* ^*11*^, and *pak* ^*myr*^ [34]; *dpix* ^*1*^ [16]; *GluRIIA* ^*SP16*^, and *Df(2L)cl* ^*h4*^ [7] (from A. DiAntonio, Washington University). The *G14-Gal4* was obtained from C. Goodman (University of California at Berkeley).

### Antibody generation and protein analysis

The rabbit polyclonal anti-Neto-β antibodies were generated against two synthetic peptides: β1 (GRSHYGGLLVTSTAKQP) and β2 (LDDVSNRYYREAVPL) (21st Century Biochemicals) and separated by affinity purification. The rabbit polyclonal anti-GluRIIB and anti-GluRIIC were generated as previously described [8] against synthetic peptides ASSAKKKKKTRRIEK, and respectively QGSGSSSGSNNAGRGEKEARV (Pacific Immunology Corp).

To analyze muscle proteins, wandering third instar larvae were dissected, and all tissues except for the body wall (muscle and cuticle) were removed. The body walls were mechanically disrupted and lysed in lysis buffer (50 mM Tris-HCl, 150 mM NaCl, 1% Triton X-100, 1% deoxycholate, protease inhibitor cocktail (Roche) for 30 min on ice. The lysates were separated by SDS-PAGE on 4%–12% NuPAGE gels (Invitrogen) and transferred onto PVDF membranes (Millipore). *Drosophila* S2 cells were used for the production of recombinant proteins [12]. Full-length cDNA for *neto-β* from RE42119 was subcloned in pAcPA-based plasmids for expression in S2 cells under the actin promoter. The Neto-β truncation (*neto* ^*203*^-like) was generated by looping out the deleted fragment using the QuickChange site-directed mutagenesis kit. All constructs were verified by DNA sequencing.

Primary antibodies were used at the following dilutions: rat anti-Neto-ex [12], 1:1000; anti-Tubulin (Sigma-Aldrich),1:1000; rabbit anti-Neto-β, 1:1000; rabbit anti-GluRIIC, 1:1000; rabbit anti-PAK, 1:5000 (a gift from Nicholas Harden) [35]; mouse anti-Dlg (4F3), 1:1000; mouse anti-V5 (Invitrogen), 1:1000. Immune complexes were visualized using secondary antibodies coupled with IR-Dye 700 or IR-Dye 800 followed by scanning with the Odyssey infrared imaging system (LI-COR Biosciences).

### Immunohistology

Wandering third instar larvae were dissected as described previously in ice-cooled Ca^2+^-free HL-3 solution [49,50]. Embryos at 18h after egg laying (AEL) were dechorinated and genotyped and, after an additional incubation of 2 h at room temperature, were dissected as described previously [12]. First instar larvae were similarly dissected within 2 h from hatching. The samples were fixed in 4% paraformaldehyde (Polysciences, Inc.) for 20 min or in Bouin’s fixative (Bio-Rad) for 3 min and washed in PBS containing 0.5% Triton X-100.

Primary antibodies from Developmental Studies Hybridoma Bank were used at the following dilutions: mouse anti-GluRIIA (8B4D2), 1:100; mouse anti-Dlg (4F3), 1:1000; mouse anti-Brp (Nc82), 1:200; mouse anti-CSP (6D6), 1:1000: mouse anti-α-Spectrin (3A9), 1:50: mouse anti-FasII (1D4),1:10. Other primary antibodies were utilized as follow: rabbit anti-PAK, 1:2000; rat anti-Neto-ex, 1:1000 [12]; and Cy5- conjugated goat anti-HRP, 1:1000 (Jackson ImmunoResearch Laboratories, Inc.). Alexa Fluor 488-, Alexa Fluor 568-, and Alexa Fluor 647- conjugated secondary antibodies (Molecular Probes) were used at 1:200. All samples were mounted in ProLong Gold (Invitrogen).

Samples of different genotypes were processed simultaneously and imaged under identical confocal settings in the same imaging session with a laser scanning confocal microscope (CarlZeiss LSM780). All images were collected as 0.2m optical sections and the *z*-stacks were analyzed with Imaris software (Bitplane). To detect positive puncta we used the spot finding Imaris algorithm followed by manual inspection and correction. To quantify fluorescence intensities synaptic ROI areas surrounding anti-HRP immunoreactivities were selected and the signals measured individually at NMJs (muscle 4, segment A3) from ten or more different larvae for each genotype (number of samples is indicated in the graph bar). The signal intensities were calculated relative to HRP volume and subsequently normalized to control. Boutons were counted in preparations double labeled with anti-HRP and anti-Dlg. All quantifications were performed while blinded to genotype. The numbers of samples analyzed are indicated inside the bars. Statistical analyses were performed using the Student t-test with a two-tailed distribution and a two-sample unequal variance. Error bars in all graphs indicate standard deviation ±SEM. ***; p<0.001, **; p<0.005, *; p<0.05, ns; p>0.05.

### Electrophysiology

The standard larval body wall muscle preparation first developed by Jan and Jan (1976) [51] was used for electrophysiological recordings [52]. Wandering third instar larvae were dissected in physiological saline HL-3 saline [49], washed, and immersed in HL-3 containing 0.8 mM Ca^2+^ or 0.3 mM Ca^2+^ using a custom microscope stage system [53]. The nerve roots were cut near the exiting site of the ventral nerve cord so that the motor nerve could be picked up by a suction electrode. Intracellular recordings were made from muscle 6. Data were used when the input resistance of the muscle was >5 MΩ and the resting membrane potential was between -60 mV and -80 mV. The input resistance of the recording microelectrode (backfilled with 3 M KCl) ranged from 20 to 25 MΩ. Muscle synaptic potentials were recorded using an Axon Clamp 2B amplifier (Axon Instruments) and pClamp 10 software. Following motor nerve stimulation with a suction electrode (100 μsec, 5 V), evoked EJPs were recorded. Four to six EJPs evoked by low frequency of stimulation (0.1 Hz) were averaged. For mini recordings, TTX (1 μM) was added to prevent evoked release [49]. To calculate mEJP mean amplitudes, 50–200 events from each muscle were measured and averaged using the Mini Analysis program (Synaptosoft). Minis with a slow rise and falling time arising from neighboring electrically coupled muscle cells were excluded from analysis [54,55]. Quantal content was calculated by dividing the mean EJP by the mean mEJP after correction of EJP amplitude for nonlinear summation according to previously described methods [56,57]. Corrected EJP amplitude = E[Ln[E/(E—recorded EJP)]], where E is the difference between reversal potential and resting potential. The reversal potential used in this correction was 0 mV [57,58]. Data are presented as mean ±SEM, unless otherwise specified; EJP amplitudes and quantal contents after the nonlinear correction are shown. Student T-test was used to assess statistically significant differences among the genotypes.

### Electron microscopy

Wandering third instar larvae were dissected in physiological saline HL-3 saline and fixed for 30 min on dissection plate in fixation buffer (0.1 M Sodium Cacodylate buffer, pH7.2; 2 mM MgCl_2_; 1% glutaraldehyde; 4% paraformaldehyde). The samples were trimmed to include only the abdominal segments A2 and A3, transferred in a 1.5mL Eppendorf tube, fixed overnight at 4°C, then washed extensively with 0.1 M Sodium Cacodylate buffer with 132 mM Sucrose, pH 7.2. The samples were further processed and analyzed according to published protocols [59] at the Microscopy and Imaging Facility, NICHD.

## Supporting Information

S1 Fig Sequence alignment for Neto-β cytoplasmic domain. ClustalW alignment of Neto-β cytoplasmic domains in several *Drosophila* species (*Drosophila melanogaster*, *Drosophila ananassae* and *Drosophila pseudoobscura*) reveals blocks of highly conserved sequences, including SH2 and SH3 binding domains, poly-H and poly-Q motifs, and putative phosphorylation sites (putative CaMKII phosphorylation site is indicated in red). The Neto-β truncated form (*neto* ^*βshort*^ allele) is predicted to retain the first 88 conserved intracellular residues followed by 8 additional, unrelated residues (shown in orange). The synthetic peptides used to generate the Neto-β isoform specific antibodies are marked by blue boxes.
(TIF)

S2 Fig Characterization of Neto-β isoform specific antibodies. (A) Western blot of lysates from S2 cells transfected with control (mock), Neto-α or Neto-β expression constructs. The Neto-ex rat polyclonal antibodies label both Neto isoforms, while the Neto-β rabbit polyclonal antibodies can only recognize the Neto-β recombinant protein. Arrowheads point to unprocessed and processed Neto-α (white) and Neto-β (black). The apparent molecular weights are higher than predicted likely due to post-translational modifications: ~100/85 kD observed for unprocessed/processed Neto-α variants (75/62 kD calculated) and ~115/100 kD for Neto-β (92/77 kD calculated). (B) Representative confocal images of NMJ4 boutons in third instar larvae labeled for Neto-β (green), HRP (blue) and Neto-ex (red) (left panels) or GluRIIA (red) (right panels). Neto-β positive puncta co-localize with Neto-ex and GluRIIA signals at synaptic sites. Scale bars: 20 μm, 2 μm in details.
(TIF)

S3 Fig The iGluRs synaptic signals at various *neto* mutant NMJs. (A) Representative confocal images of NMJ4 boutons in third instar larvae of indicated genotypes labeled for Neto-ex (red), GluRIIC (green) and HRP (blue). *neto-β* mutant NMJs have progressively reduced levels of Neto-ex positive synaptic signals (quantified relative to HRP in B). The levels of synaptic Neto closely match the GluRIIC synaptic signals. (C-D) Representative confocal images of NMJ4 boutons in third instar larvae of control (precise excision for *neto-β* allelic series), *w* ^*1118*^ and *neto* ^*hypo*^ labeled for HRP (blue), and Brp (red), GluRIIC (green) (C) or GluRIIA (red), GluRIIB (green) (D). The iGluRs signals are barely detectable at *neto* ^*hypo*^ NMJs when imaged side-by-side with the precise excision and with *w* ^*1118*^, the closest control for the *neto* hypomorphs. (E) Table summarizing the quantifications from the experiments presented above and in Fig 4. Error bars indicate SEM. ***; p<0.001. Scale bars: 20 μm.
(TIF)

S4 Fig Recruitment of postsynaptic components at *neto-β* mutant NMJs is rescued by a duplication covering the *neto* gene. Representative confocal images of NMJ4 boutons (segment A3) in third instar larvae of indicated genotypes labeled for PAK (red), Dlg (green) and Neto-ex (blue). The synaptic accumulation of PAK and Dlg is restored at *neto-β* mutant NMJs by a duplication covering the *neto* locus. Genotypes: control (precise excision), *neto* ^*βshort*^, *neto* ^*βshort*^;*;Dp (neto* ^*βshort*^ */Y;;Dp(1;3)DC270/+)*, *neto* ^*βnull*^, and *neto* ^*βnull*^;*;Dp (neto* ^*βnull*^ */Y;;Dp(1;3)DC270/+)*. Scale bars: 20μm, 2μm in details.
(TIF)

S5 Fig CSP and -Spectrin have normal distribution at *neto-β* mutant NMJs. (A–B) Confocal images of NMJ4 boutons (segment A3) in third instar larvae of indicated genotypes labeled for HRP (blue), and Cystein string protein (CSP) (green)(A) or -Spectrin (green) (B). CSP and -Spectrin localize normally at *neto-β* mutant NMJs. Scale bars: 20μm, 2μm in details.
(TIF)

S6 Fig The synaptic localization of Neto does not require dPix. Confocal images of NMJ4 boutons (segment A3) in third instar larvae labeled for Neto-ex (red), PAK (green), and HRP (blue). The Neto-positive synaptic signals but not PAK signals are present at *dpix* mutant NMJs. The Neto-ex staining is less uniform than in control (*w* ^*1118*^) presumably because of generally altered NMJ morphology in *dpix* mutant larvae. Scale bars: 2μm.
(TIF)

S7 Fig Neto- alone cannot ensure a normal GluRIIA/GluRIIB ratio at the PSD. Confocal images of NMJ4 boutons (segment A3) in third instar larvae labeled for GluRIIA (red), GluRIIB (green), and HRP (blue) in the control and the *neto* ^*null*^;*G14>neto* ^*α low*^ (*neto* ^*36*^ */Y*; *G14-Gal4/UAS-neto-A9*, reared at 25°C). These animals show a significant reduction of the GluRIIA synaptic levels (to 40% of control) and a more variable increase in GluRIIB signals. Error bars indicate SEM. **; p<0.005, ns; p>0.05. Scale bars: 20μm.
(TIF)

S1 Table Summary of physiology defects at *neto-β* mutant NMJs. (TIF)
