# Supplementary figures and images for: Neto-Mediated Intracellular Interactions Shape Postsynaptic Composition at the Drosophila Neuromuscular Junction
Source: PLoS Genet. 2015 Apr 23;11(4):e1005191. doi: 10.1371/journal.pgen.1005191 (PMC4408064; doi:10.1371/journal.pgen.1005191)

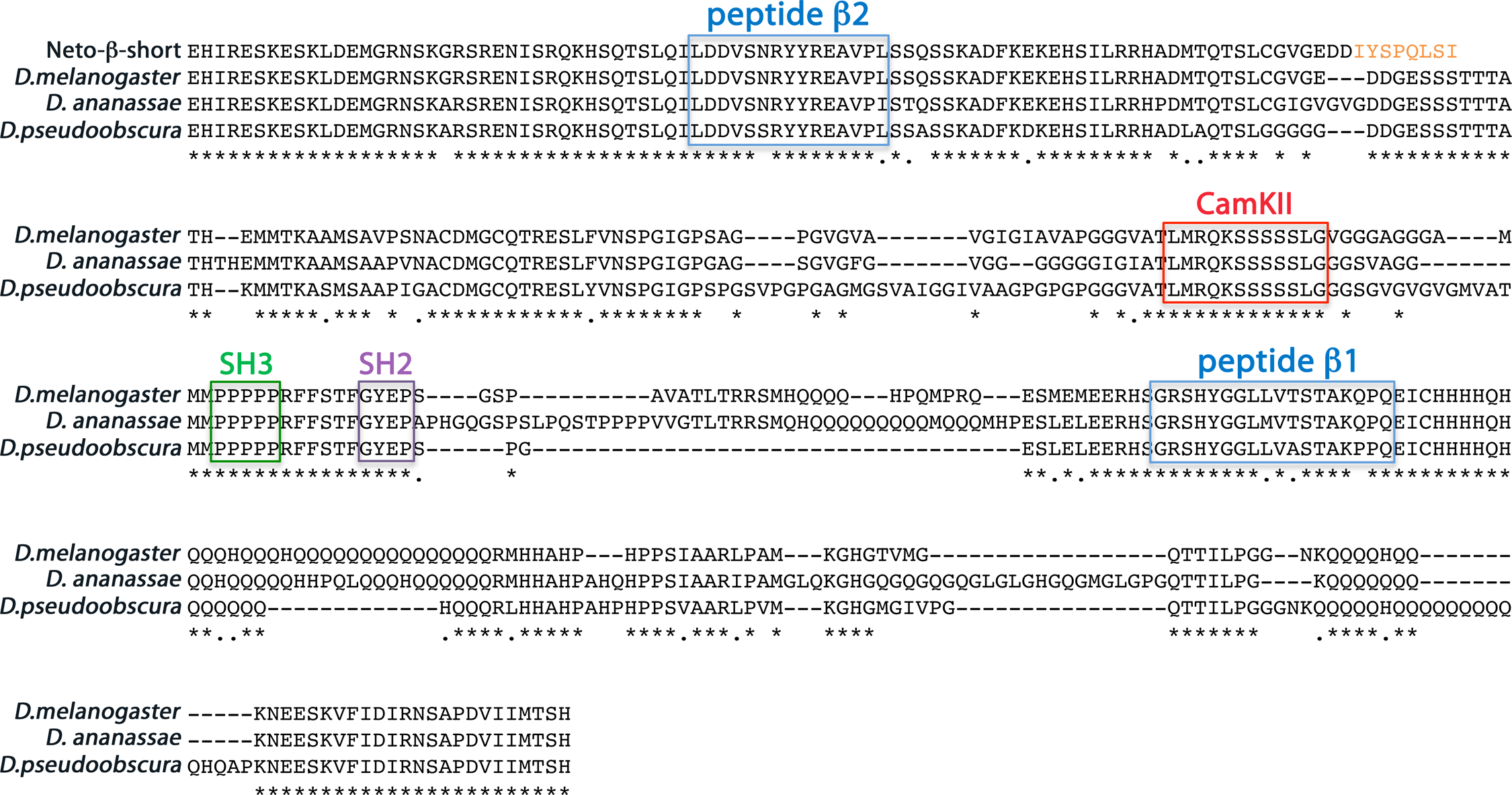

Supplement: S1 Fig — ClustalW alignment of Neto-β cytoplasmic domains in several Drosophila species (Drosophila melanogaster, Drosophila ananassae and Drosophila pseudoobscura) reveals blocks of highly conserved sequences, including SH2 and SH3 binding domains, poly-H and poly-Q motifs, and putative phosphorylation sites (putative CaMKII phosphorylation site is indicated in red). The Neto-β truncated form (neto βshort allele) is predicted to retain the first 88 conserved intracellular residues followed by 8 additional, unrelated residues (shown in orange). The synthetic peptides used to generate the Neto-β isoform specific antibodies are marked by blue boxes. (TIF) [file pgen.1005191.s001.tif]

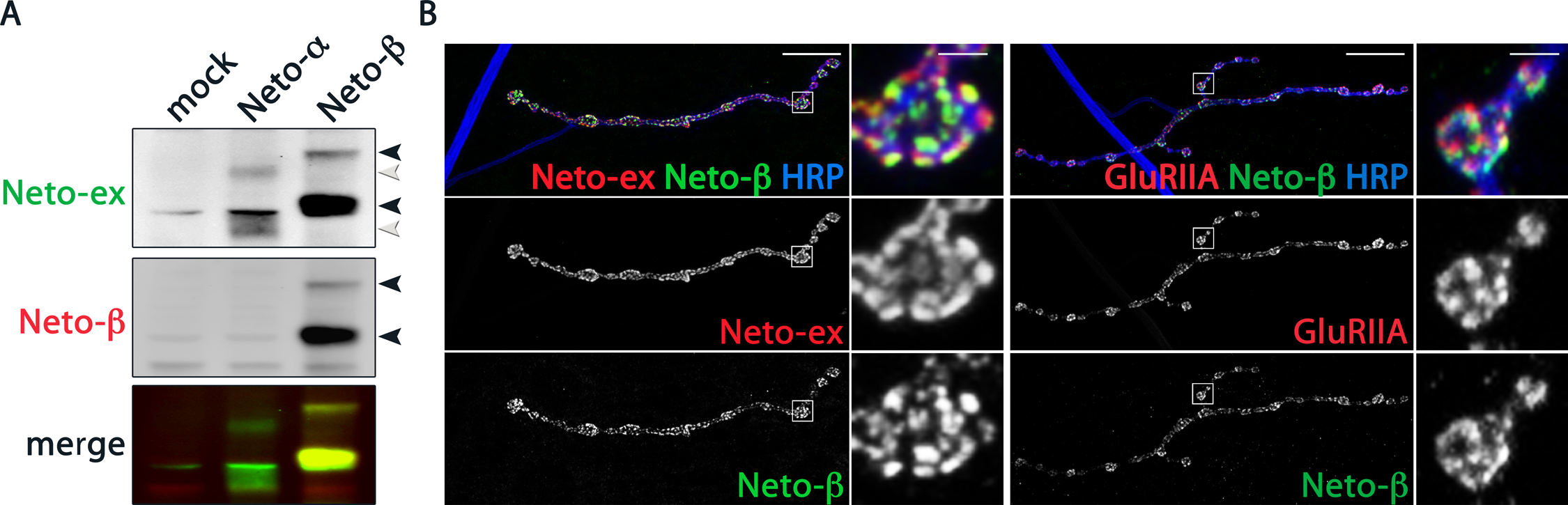

Supplement: S2 Fig — (A) Western blot of lysates from S2 cells transfected with control (mock), Neto-α or Neto-β expression constructs. The Neto-ex rat polyclonal antibodies label both Neto isoforms, while the Neto-β rabbit polyclonal antibodies can only recognize the Neto-β recombinant protein. Arrowheads point to unprocessed and processed Neto-α (white) and Neto-β (black). The apparent molecular weights are higher than predicted likely due to post-translational modifications: ~100/85 kD observed for unprocessed/processed Neto-α variants (75/62 kD calculated) and ~115/100 kD for Neto-β (92/77 kD calculated). (B) Representative confocal images of NMJ4 boutons in third instar larvae labeled for Neto-β (green), HRP (blue) and Neto-ex (red) (left panels) or GluRIIA (red) (right panels). Neto-β positive puncta co-localize with Neto-ex and GluRIIA signals at synaptic sites. Scale bars: 20 μm, 2 μm in details. (TIF) [file pgen.1005191.s002.tif]

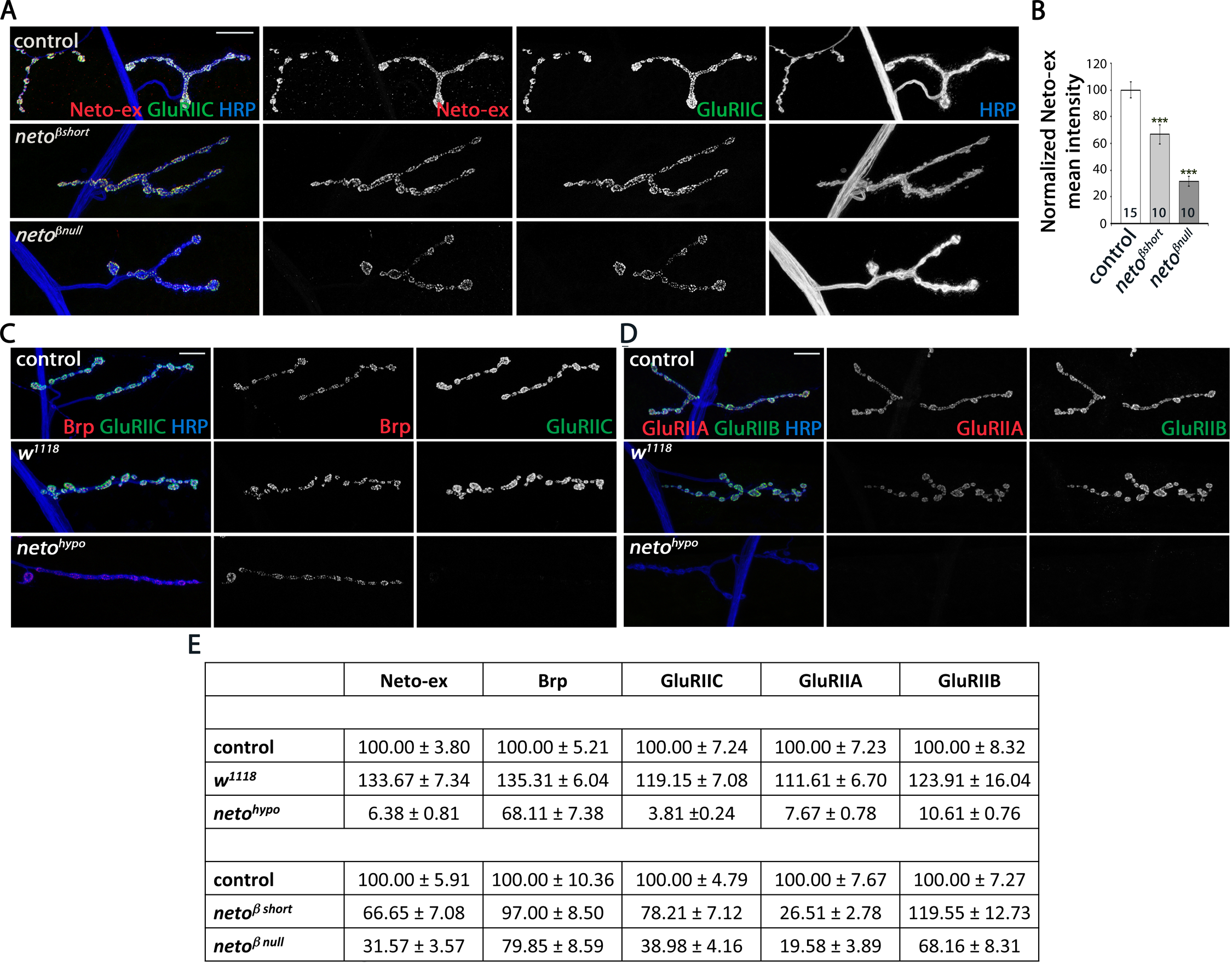

Supplement: S3 Fig — (A) Representative confocal images of NMJ4 boutons in third instar larvae of indicated genotypes labeled for Neto-ex (red), GluRIIC (green) and HRP (blue). neto-β mutant NMJs have progressively reduced levels of Neto-ex positive synaptic signals (quantified relative to HRP in B). The levels of synaptic Neto closely match the GluRIIC synaptic signals. (C-D) Representative confocal images of NMJ4 boutons in third instar larvae of control (precise excision for neto-β allelic series), w 1118 and neto hypo labeled for HRP (blue), and Brp (red), GluRIIC (green) (C) or GluRIIA (red), GluRIIB (green) (D). The iGluRs signals are barely detectable at neto hypo NMJs when imaged side-by-side with the precise excision and with w 1118, the closest control for the neto hypomorphs. (E) Table summarizing the quantifications from the experiments presented above and in Fig 4. Error bars indicate SEM. ***; p<0.001. Scale bars: 20 μm. (TIF) [file pgen.1005191.s003.tif]

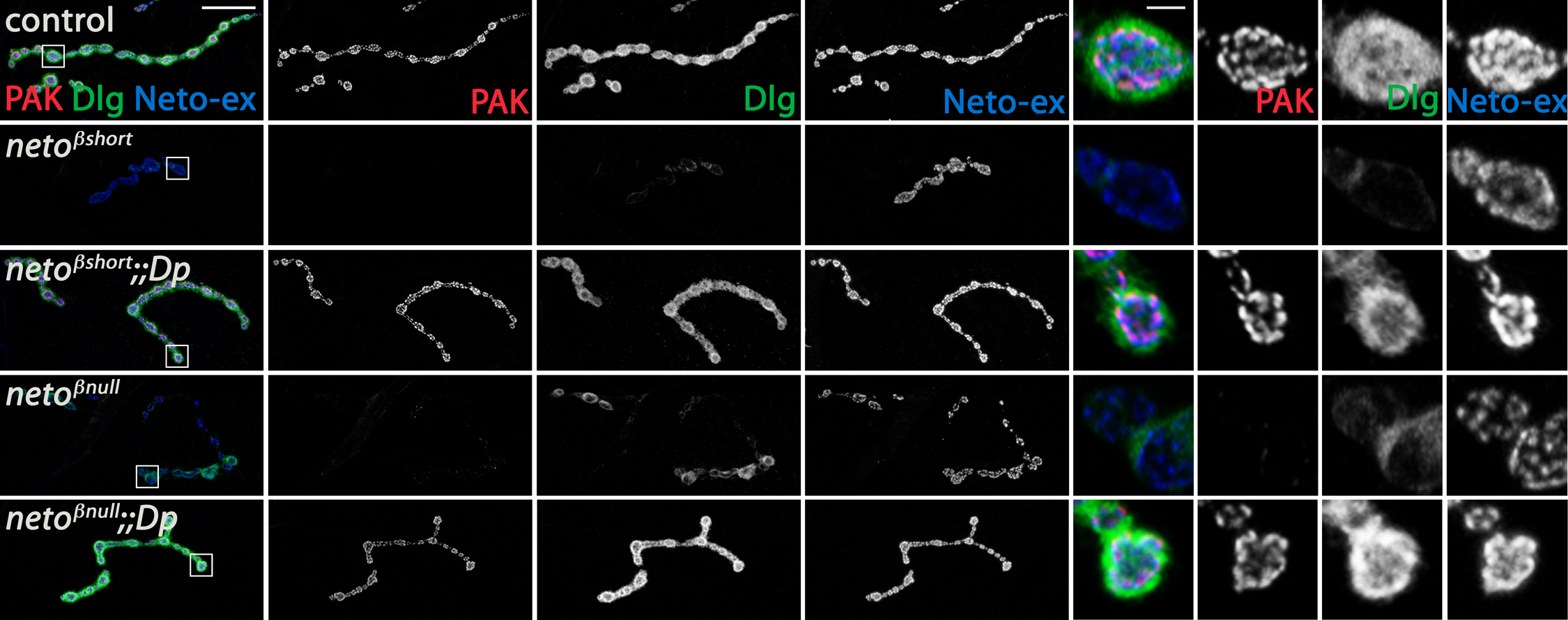

Supplement: S4 Fig — Representative confocal images of NMJ4 boutons (segment A3) in third instar larvae of indicated genotypes labeled for PAK (red), Dlg (green) and Neto-ex (blue). The synaptic accumulation of PAK and Dlg is restored at neto-β mutant NMJs by a duplication covering the neto locus. Genotypes: control (precise excision), neto βshort, neto βshort;;Dp (neto βshort /Y;;Dp(1;3)DC270/+), neto βnull, and neto βnull;;Dp (neto βnull /Y;;Dp(1;3)DC270/+). Scale bars: 20μm, 2μm in details. (TIF) [file pgen.1005191.s004.tif]

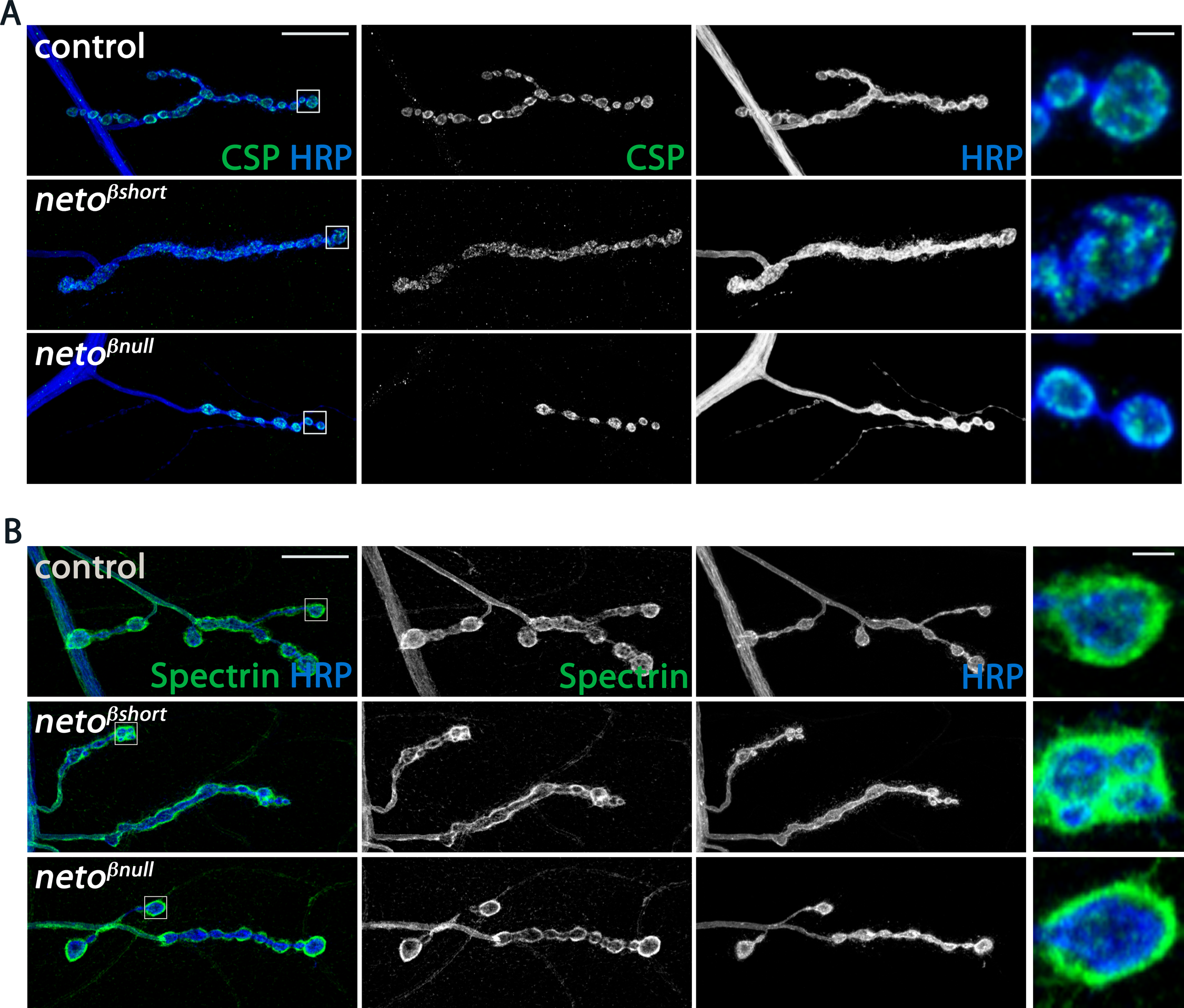

Supplement: S5 Fig — (A–B) Confocal images of NMJ4 boutons (segment A3) in third instar larvae of indicated genotypes labeled for HRP (blue), and Cystein string protein (CSP) (green)(A) or -Spectrin (green) (B). CSP and -Spectrin localize normally at neto-β mutant NMJs. Scale bars: 20μm, 2μm in details. (TIF) [file pgen.1005191.s005.tif]

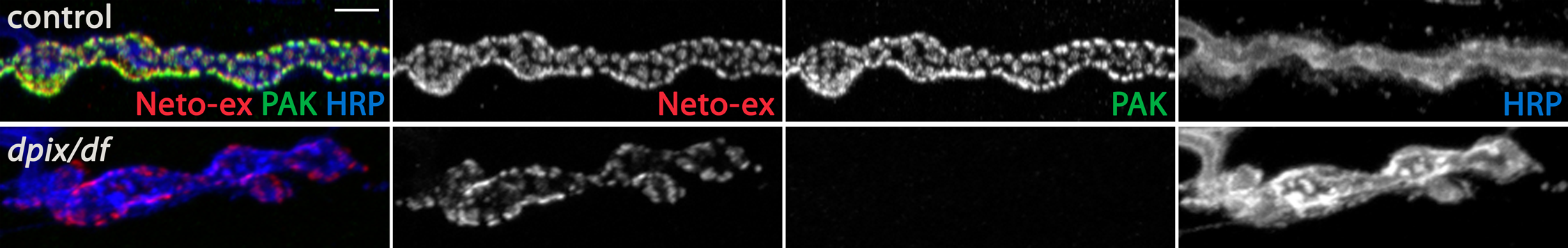

Supplement: S6 Fig — Confocal images of NMJ4 boutons (segment A3) in third instar larvae labeled for Neto-ex (red), PAK (green), and HRP (blue). The Neto-positive synaptic signals but not PAK signals are present at dpix mutant NMJs. The Neto-ex staining is less uniform than in control (w 1118) presumably because of generally altered NMJ morphology in dpix mutant larvae. Scale bars: 2μm. (TIF) [file pgen.1005191.s006.tif]

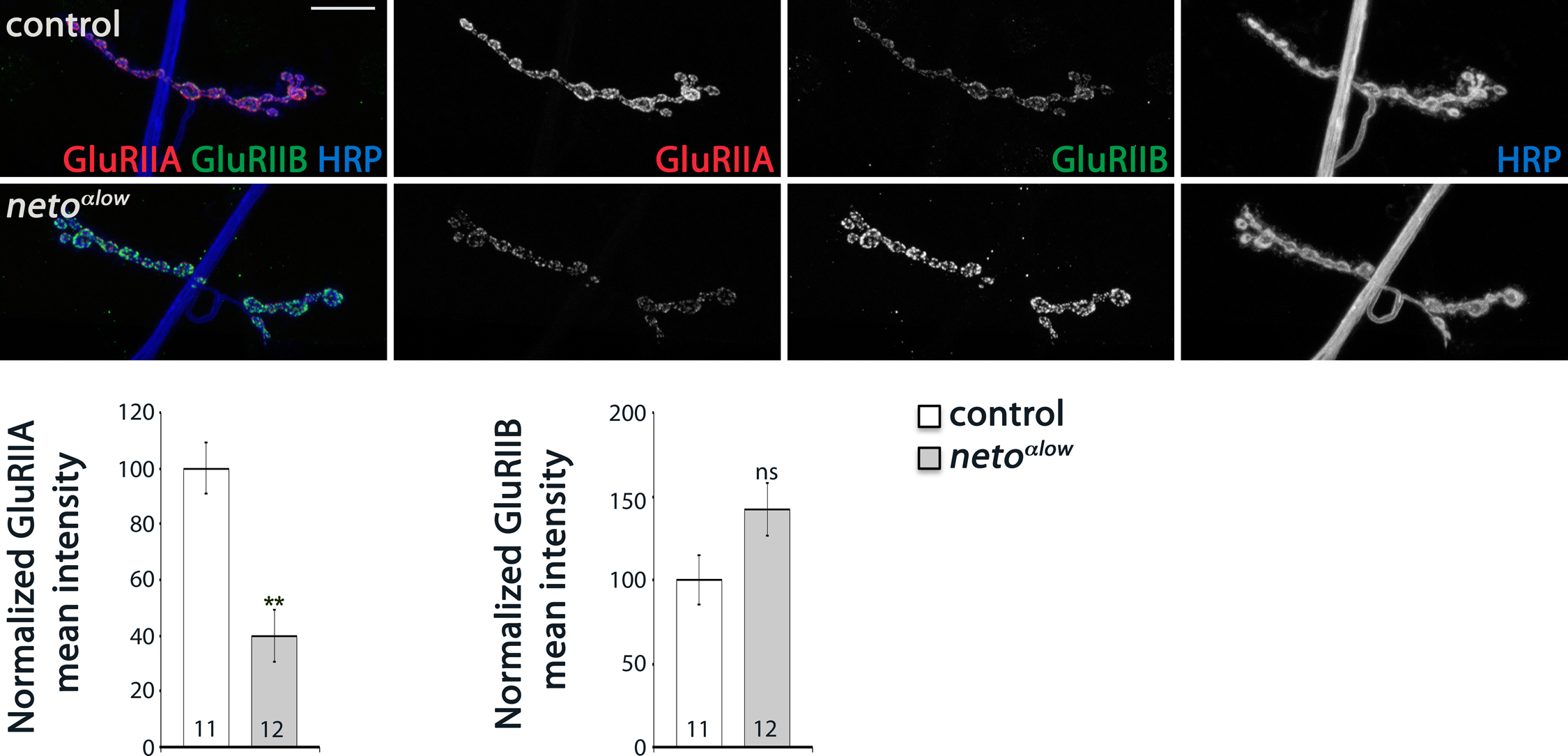

Supplement: S7 Fig — Confocal images of NMJ4 boutons (segment A3) in third instar larvae labeled for GluRIIA (red), GluRIIB (green), and HRP (blue) in the control and the neto null;G14>neto α low (neto 36 /Y; G14-Gal4/UAS-neto-A9, reared at 25°C). These animals show a significant reduction of the GluRIIA synaptic levels (to 40% of control) and a more variable increase in GluRIIB signals. Error bars indicate SEM. **; p<0.005, ns; p>0.05. Scale bars: 20μm. (TIF) [file pgen.1005191.s007.tif]

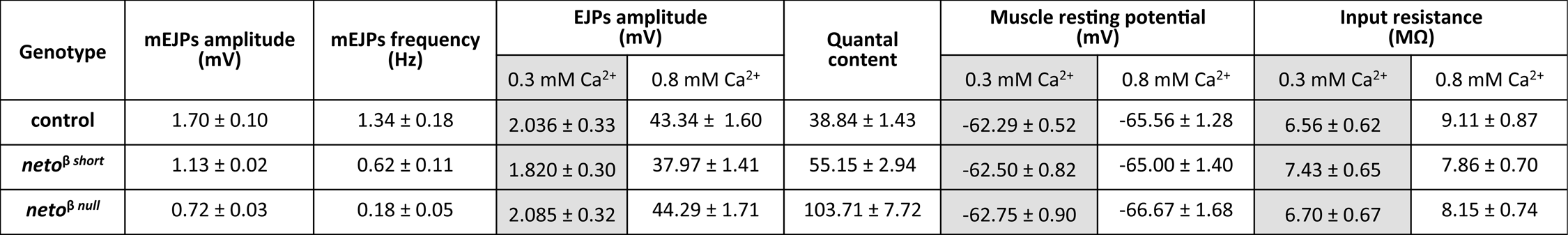

Supplement: S1 Table — (TIF) [file pgen.1005191.s008.tif]
